# A socio-environmental geodatabase for integrative research in the transboundary Rio Grande/Río Bravo basin

**DOI:** 10.1038/s41597-020-0410-1

**Published:** 2020-03-06

**Authors:** Sophie Plassin, Jennifer Koch, Stephanie Paladino, Jack R. Friedman, Kyndra Spencer, Kellie B. Vaché

**Affiliations:** 1 University of Oklahoma, Department of Geography and Environmental Sustainability, 100 E Boyd St, Suite 510, Norman, OK 73019 USA; 2 MeroLek Research, PO Box 973, Athens, 30603 GA USA; 3 University of Oklahoma, Center for Applied Social Research, 5 Partners Place, 201 Stephenson Parkway, Suite 4100, Norman, 73019 OK USA; 4 Oregon State University, Biological and Ecological Engineering, 124 SW 26th Street, Gilmore Hall 232, Corvallis, 97331 OR USA

**Keywords:** Environmental impact, Hydrology, Environmental impact

## Abstract

Integrative research on water resources requires a wide range of socio-environmental datasets to better understand human-water interactions and inform decision-making. However, in transboundary watersheds, integrating cross-disciplinary and multinational datasets is a daunting task due to the disparity of data sources and the inconsistencies in data format, content, resolution, and language. This paper introduces a socio-environmental geodatabase that transcends political and disciplinary boundaries in the Rio Grande/Río Bravo basin (RGB). The geodatabase aggregates 145 GIS data layers on five main themes: (i) Water & Land Governance, (ii) Hydrology, (iii) Water Use & Hydraulic Infrastructures, (iv) Socio-Economics, and (v) Biophysical Environment. Datasets were primarily collected from public open-access data sources, processed with ArcGIS, and documented through the FGCD metadata standard. By synthesizing a broad array of datasets and mapping public and private water governance, we expect to advance interdisciplinary research in the RGB, provide a replicable approach to dataset compilation for transboundary watersheds, and ultimately foster transboundary collaboration for sustainable resource management.

## Abstract (summary)

**Table:** 

Measurement(s)	water resources • Socioeconomic Factors • Infrastructure • Environment
Technology Type(s)	digital curation
Sample Characteristic - Environment	watershed • socio-environmental system
Sample Characteristic - Location	Rio Grande • Rio Bravo

Machine-accessible metadata file describing the reported data: 10.6084/m9.figshare.11807424

## Background & Summary

Finding solutions to manage scarce water resources to meet human water needs while sustaining ecosystems has become a priority for research^1–4^ and policy makers. In 2015, the United Nations (UN) adopted 17 Sustainable Development Goals (SDG), including “Ensuring access to water and sanitation for all” (SDG6)^5^. However, increasing pressure on water resources, due to population growth and climate change, may be an obstacle to reaching this goal and may lead to increased competition and political tensions. This especially applies to transboundary river systems^6^, which represent 60% of freshwater supply basins and almost 50% of the total land area on Earth^7^.

In transboundary basins, data- and information-sharing provide a mechanism to foster cooperation among countries and, ultimately, achieve more sustainable water management and maintain peace and security^7^. Synthesizing a broad range of environmental and social datasets is further critical for in-depth understanding of watershed dynamics^8^ and for assessing the sustainability of water allocation^9^. In large areas, spatial datasets are also valuable to capture the spatial heterogeneity of local conditions and for better understanding of different development trends across a region^10^. However, integrating scattered cross-disciplinary and multinational datasets is challenged by the disparity of data sources, transboundary discontinuity^11^ and the inconsistencies in format, content, spatial and temporal resolution, languages^12^, and institutional norms. This can limit the study of transboundary socio-environmental systems, and calls for the development of a harmonized, transboundary, open-access database.

The Rio Grande/Río Bravo basin (RGB) epitomizes the challenges of managing water resources under conditions of scarcity^13^ and transboundary cooperation issues^14^. Covering 552,382 km^2^, the basin is shared by two countries (United States and Mexico) and eight states, three in the U.S. (Colorado, New Mexico, and Texas) and five in Mexico (Chihuahua, Coahuila, Durango, Nuevo León, and Tamaulipas). Distribution of water resources is governed by distinct national and state water rights regimes, as well as bi-national and inter-state agreements^14^. The river is highly engineered via extensive damming and channelization^15^, which significantly disturb the natural flow regime^16^. Classified as one of the most endangered in the world^17^, the river hosts several endangered and threatened species, such as the Rio Grande Silvery Minnow, and the Southwestern Willow Flycatcher. Despite chronically severe water scarcity, human water demand has grown along with an increase in population (current population is estimated at 10.5 million people^18^). Furthermore, irrigated agriculture makes up approximately 83.5% of the total surface and groundwater withdrawals^19,20^ while cultivated areas cover only 3.5% of the basin^21^. Projected climate change and population growth are likely to lead to a growing imbalance between water supply and demand, potentially impacting the sustainability of the basin^22^ and resulting in a cascade of negative impacts (e.g., increased risk of wildfires)^23^.

To overcome the challenges of data- and information sharing in transboundary basins, several initiatives were launched in recent years. The Transboundary Freshwater Dispute Database gathers global and regional information for the world’s 310 international river basins, including the RGB^24^. Examples of topics include population, land-cover, irrigation, rainfall, and dams (http://gis.nacse.org/tfdd/index.php). In the RGB, a basin level database synthesizing hydrologic, administrative, land-use/cover, and water management datasets, was also developed at a finer resolution^25,26^. However, we are not aware of a similar approach to capture the social heterogeneity and complexity across the basin^27^. Hence, we compiled a comprehensive socio-environmental geodatabase for the RGB, encompassing geospatial data sets related to *Water & Land Governance*, *Hydrology, Water Use & Hydraulic Infrastructures*, *Socio-Economics*, and *Biophysical Environment*. An innovative contribution of our geodatabase is to provide open-access to a thorough collection of reliable and well documented, basin-wide geospatial data sets, as well as, to our knowledge, the most extensive spatial picture of multi-scale water governance in the RGB. The geodatabase expands the availability of information detailing the social and environmental characteristics of the basin and enables an integrated and cross-disciplinary approach to the watershed. Many outputs can be derived from the geodatabase, including maps, spatially explicit integrated models, and statistical analyses (see *Usage Notes* for additional details). The data synthesis approach presented here is transferable to other transboundary basins, and the geodatabase will help the research, management, and policy-making communities to foster transboundary collaboration.

## Methods

### Dataset preparation

**Fig. 1:**
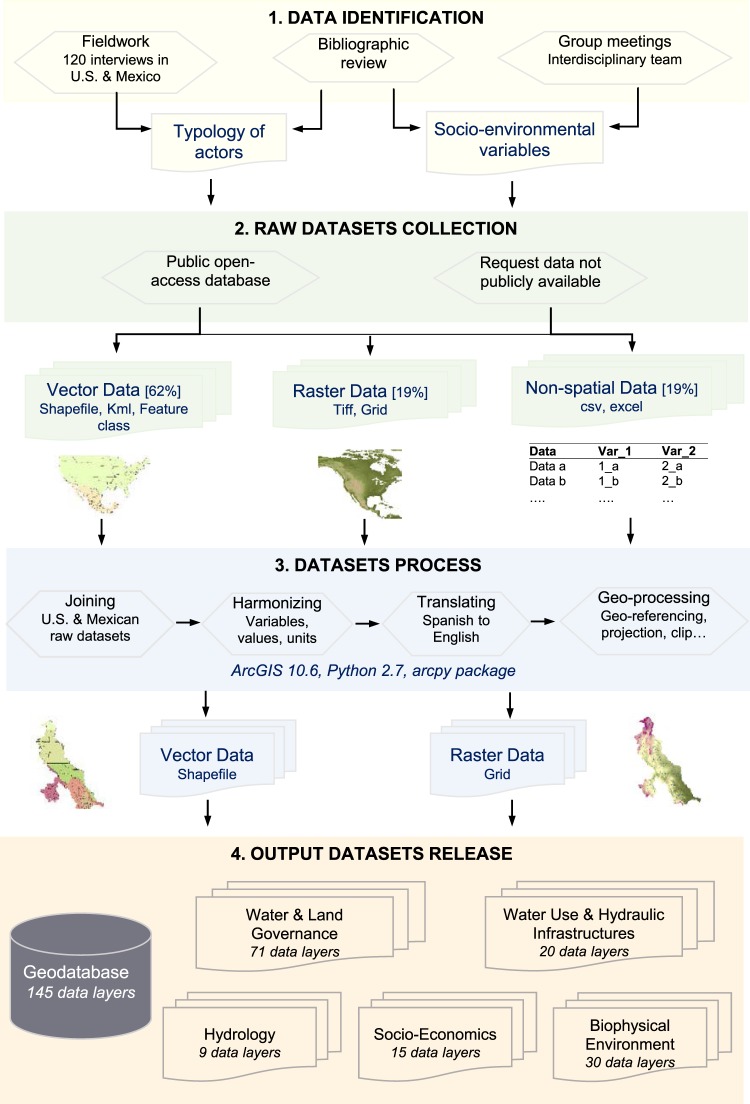
Schematic overview of the workflow applied to produce the socio-environmental geodatabase for the Rio Grande/Río Bravo basin.

The development of the socio-environmental geodatabase followed four steps: (1) data identification; (2) collection of raw data; (3) assembling, harmonizing, and geoprocessing of datasets; and (4) organization of the final geospatial data sets into the geodatabase (Fig. 1).

First, we drew on an interdisciplinary collaboration among social and environmental scientists to identify critical data requirements to support socio-environmental research in the RGB. The environmental anthropologists shared primary data analysis from ethnographic research and developed a qualitative typology of actors that we used to identify water and land governance datasets. We integrated land management as a key component for the study of socio-environmental dynamics in the watershed because land management decisions affect water run-off, stream flow, aquifer recharge, and soil erosion. The actor typology distinguishes the actors according to their roles in water and land management (water supply, consumption, infrastructure management, environmental protection, recreation) and their spatial scale of action (from local to bi-national level; Supplementary Information 1).

Second, we gathered 210 raw datasets from 49 distinct sources totalling around 53 GB. Where possible, and if the spatial resolution was high enough, we prioritized global datasets over national datasets and national datasets over regional datasets to limit data inconsistency and heterogeneity. Most of the raw datasets were collected from national agencies (67%), followed by state agencies (13%), global agencies (2%) and binational agencies (7%). We also collected datasets from joint research projects (5%) and private institutions (6%). Most of the input datasets were available open-access online. We also contacted public or private institutions to access unpublished information and were granted permission to publish the synthesized data. The original formatting of the raw datasets varied greatly. 62% were vector data (51.5% shapefiles, 3% KML/KMZ files, 7.5% feature class), 19% were raster files, and 19% were only available in tabulated format (comma-delimited text or Microsoft Excel files). In terms of language, 73% of the raw datasets were in English and 27% in Spanish. The detailed list of raw datasets used to produce the geospatial output datasets is available in Supplementary Information 2. For each raw dataset, we included a short description, the spatial and temporal dimensions, the format, language, data source, and date of access.

Third, we assembled, harmonized, and geoprocessed the datasets using ESRI’s ArcGIS 10.6 and, where appropriate, the Python programming language (version 2.7) and the arcpy package for automating the geoprocessing workflow. Geoprocessing operations varied according to the type of raw dataset, and a detailed description of these operations is available in the metadata for each geographic layer. In general, we tried to synthesize scattered state and national raw data into one data layer. Where necessary, we harmonized the information in the datasets (variables, values, units of measurement) and translated Spanish information to English. Geospatial operations included the projection of the final datasets to the most appropriate projected coordinate system that minimized areal distortion for the whole RGB (North America Albers Equal Area Conic), and clipping datasets to the RGB boundary. Instead of using the hydrological basin boundary, we used the spatial boundaries of the Rio Grande/Río Bravo socio-environmental system as delineated by Koch *et al*.^27^. For all political jurisdictional boundary information (national, state, or county), given the spatial mismatch with the catchment boundary, we prioritized the use of administrative boundaries over catchment boundaries in locations where they overlap.

Fourth, we stored the resulting 145 geographic layers, including 125 vector data and 20 raster data (GRID), in a geodatabase with a total size of 1.40 GB. We organized the datasets into five main themes: *Water & Land Governance*, *Hydrology, Water Use & Hydraulic Infrastructures*, *Socio-Economics*, and *Biophysical Environment*.

### Metadata

For each individual data layer, we produced a standardized metadata record, following the Federal Geographic Data Committee Content Standard for Digital Geospatial Metadata (FGDC CSDGM). The standard includes seven types of information: Identification Information (basic information about the dataset such as the citation, description, spatial domain, access constraints, etc.), Data Quality Information, Spatial Data Organization Information, Spatial Reference Information (Geographic Coordinate System Definition), Entity and Attribute Information, Distribution Information, and the Metadata Reference Information. The metadata records have been created through the ArcMap graphical user interface and are automatically included with the hosted layer item. The user can export the metadata in XML format through the ArcMap graphical user interface if needed.

## Data Records

The socio-environmental geodatabase is freely and publicly available on the OSF repository^28^. The collection of datasets can be downloaded as a single file (.gdb) or as individual geospatial output layers (shapefiles or raster files). Here, we describe the geospatial datasets available for the five themes *Water & Land Governance*, *Hydrology, Water Use & Hydraulic Infrastructures*, *Socio-Economics*, and *Biophysical Environment*. Online-only Table 1 and Tables 1–4 provide a systematic overview of the individual layers, i.e., the name, description, format and data sources for each theme. A comprehensive list of acronyms is included in Supplementary Information 3.

**Table 1:** List of geospatial layers for the *Hydrology* category including their description, format and input data sources.

Datasets	Output geospatial layers	Description	Format	Input source
Watershed boundary	Sub_Basins RGB_Basin	Watershed boundaries	polygon	FAO^34^
River network	Rivers	River network	line	FAO^35^
Aquifer boundaries	Aquifer	Aquifer boundaries in U.S. and Mexico	polygon	USGS^36^, CONAGUA^37^
Gauging stations	1. Gauges_usgs 2. Gauges_ibwc 3. Gauges_conagua	1. Gauges operated by USGS in U.S. 2. Gauges operated by IBWC along the border 3. Gauges operated by CONAGUA in MX	points	USGS^38^, USIBWC^39^, CONAGUA^40^
Quality Stations	Quality_Stations	Quality stations managed by IBWC and TCEQ	point	USIBWC^39^

**Table 2:** List of geospatial layers for the *Water Use & Hydraulic Infrastructures* category including their description, format and input data sources.

Datasets	Output geospatial layers	Description	Format	Input source
Withdrawals	Withdrawals_2015	Amount of surface water and groundwater used in mcm by the different sectors on a county/*municipio* basis in 2015	polygon	USGS^41^, CONAGUA^37^
Irrigation	1. Irrigation_US 2. Irrigation_MX	Spatial coverage of irrigated lands and irrigation systems (surface, sprinkler micro-irrigation, other) on a county/*municipio* basis	polygon	USGS^41^, INEGI^42^
Water Rights	1. CO_WaterRights 2. NM_WaterRights 3. TX_WaterRights 4. COA_WaterRights 5. CHI_WaterRights 6. DUR_WaterRights 7. NVL_WaterRights 8. TAM_WaterRights	1. Surface and Ground Water rights in Colorado (Division 3) 2. Surface and Ground Water rights in New Mexico 3. Surface Water rights in Texas 4. Surface water & Ground Water rights in Coahuila 5. Surface water & Ground Water rights in Chihuahua 6. Surface water & Ground Water rights in Durango 7. Surface water & Ground Water rights in Nuevo León 8. Surface water & Ground Water rights in Tamaulipas	point	CDWR^43^, NMOSE^44^, TCEQ^45^, CONAGUA^46^
Dams	1. Dams_us 2. Dams_mx	1. All dams in U.S. derived from USACE-NID 2. All dams in MX derived from CENAPRED	point	USACE^47^, CENAPRED^48^
Water diversion structures	1. Diversion_US 2. Diversion_MX 3. Diversion_CO	1. Water diversion (canal/ditch, connector, pipeline, underground conduit) in U.S. 2. Water diversion (canals, aqueducts) in MX 3. Canals in CO	line	USGS-NHD^49^, INEGI^50^, CWCB/DWR^51^
Wells	1. Wells_us_nhd 2. Wells_co_cwcb 3. Wells_nm_nmose 4. Wells_tx_twdb	1. Wells in U.S. derived from USNHD 2. Wells in CO compiled by CWCB/DWR 3. Wells in NM compiled by NMOSE 4. Wells in TX compiled by TWDB	point	USGS-NHD^49^, CWCB/DWR^51^, NMOSE^44^, TWDB^52^

**Table 3:** List of provided geospatial layers for the *Socio-Economics* category including their description, format and input data sources.

Category	Output geospatial layers	Description	Format	Input source
Population	Population	Population in 1990, 2000, 2010 [county/*municipio-*based]	polygon	US Census Bureau^53^, INEGI^54^
Population density	popdens_00 popdens_05 popdens_10 popdens_15 popdens_20	Population density in 2000, 2005, 2010, 2015, 2020 [30 arc-second resolution]	raster	CIESIN - Columbia University^55^
Income	1. Income_US 2. DistributionIncome_MX90 3. DistributionIncome_MX00 4. DistributionIncome_MX10	1. Personal income in U.S. from 1969 to 2015 [county-based] 2.3.4. Income Distribution in MX [*municipio-*based] in 1990, 2000, 2010	polygon	USBEA^56^, INEGI compiled by CONABIO^57^
Number and size of farms	1. Farms_US2007 2. Farms_US2012 3. Farms_MX2007	1.2. Number of farms, farm area (in hectares and acres) and farm area distributed by farm size in US, in 2007–2012 [county-based] 3. Number of farms and farm areas (hectares) in MX in 2007 [*municipio-*based]	polygon	USDA-NASS^58^, INEGI^42^
Transport Infrastructures	1. Roads 2. Railroads	1. Roads 2. Railroads	line	CIESIN - Columbia University/ITOS - University of Georgia^59^, INEGI/NRCan/USGS/CEC^60^

**Table 4:** List of provided geospatial layers for the Biophysical Environment category including their description, format and input data sources.

Datasets	Output geospatial layers	Description	Format	Input source
Ecoregions	1. NA_ECO_3 2. US_ECO_4	1. Ecoregions Level III for U.S. and Mexico 2. Ecoregions Level IV for U.S.	polygon	US-EPA^61,62^
Habitat	1. US_Critbab_Line, US_Crithab_Poly 2. US_Riparian 3. US_Wetlands 4. MX_Ramsar_Site 5. MX_SAP 6. MX_SPR	1. U.S. FWS Threatened & Endangered Species Active Critical Habitat Boundaries 2. Riparian areas in the U.S. 3. Wetlands in the U.S. 4. Ramsar sites in Mexico 5. Priority Focus Sites for Biodiversity Conservation in Mexico 6. Priority Sites for Restoration in Mexico	polygon, line	USFWS^63–65^, CONANP^66^, CONABIO^67,68^
Soil Cover	soil	Soil units based on FAO taxonomy	polygon	FAO^69,70^
Elevation	elev	Elevation derived from the SRTM DEM and the USGS GTOPO30 [30 arc-second resolution]	raster	FAO/IIASA/ISRIC/ISS-CAS/JRC^71^
Slope	1. slopedeg 2. slopepct	1. Slope in degree generated from the elevation 2. Slope in percent generated from the elevation	raster	FAO/IIASA/ISRIC/ISS-CAS/JRC^71^
Land Cover	lc2010	Land-cover in 2010 [30 m resolution]	raster	CCRS/CCMEO/NRCan/CONABIO/CONAFOR/INEGI/USGS^72^
Land Use	1. cdl2008, cdl2009, cdl2010, cdl2011, cdl2012, cdl2013, cdl2014, cdl2015, cdl2016, cdl2017, cdl2018 2. MX_LU_2011, MX_LU_2014 3. MX_ancrop_winter07, MX_ancrop_spring07, MX_percrop07	1. Time-series (2008–2018) of the Cropland Data Layer in US [30 m resolution] 2. Land-Use in MX in 2011 and 2014 [30 m resolution] 3. Annual Winter, Annual Spring and Perennial Areas Planted in MX in 2007 [*municipio-*based]	raster, polygon	USDA-NASS^73^, INEGI^74,75^, INEGI^42^

### Water & Land Governance (GOV)

The Water & Land Governance category is divided into 12 sub-categories, representing political jurisdiction boundaries, surface water management agencies (binational, federal, state), state and interstate multi-stakeholder platforms, groundwater-focused institutions, irrigation and conservation organizations, land management, and border control. A description of each entity with their missions is provided in Supplementary Information 1.

#### Political jurisdiction boundaries

The geodatabase provides the boundaries of four political jurisdiction divisions: nation, state, county/*municipio*, and places (cities, towns and villages). The geodatabase also provides a point dataset of the most populated places in both countries, which includes the main U.S.-MX sister cities along the border.

#### Binational surface water management agencies

The geodatabase gathers 13 vector datasets of binational infrastructure that spatializes the joint operations of the U.S. International Boundary and Water Commission (IBWC) and its Mexican counterpart the Comisión Internacional de Límites y Aguas (CILA), regarding national allocation of water, dam and reservoir operations, river course management (the banks, the berms, the vegetation), water quality, sanitation, and flood control in the border region.

#### Federal surface water management agencies

This sub-category includes two datasets of federal dams. One gathers all federal dams operated by the Comisión Nacional del Agua (CONAGUA) in Mexico. The other maps all dams owned by a federal agency in the U.S., including the U.S. Army Corps of Engineers (USACE), the Bureau of Indian Affairs (BIA), the Bureau of Land Management (BLM), the Bureau of Reclamation (USBR), the U.S. Fish and Wildlife Service (USFWS) and the U.S. Forest Service (USFS).

#### State and intrastate surface water management agencies

This sub-category depicts the boundaries of the Offices of the State Engineer (OSE) and their regional offices (divisions and districts) in charge of the administration of water rights and interstate compacts in the U.S. For Mexico, we generated spatial layers representing state-level offices of the federal agency CONAGUA. We also included entities that engage in planning, coordination, and oversight of domestic water supply and wastewater treatment state-wide: in Chihuahua, the *Junta Central de Agua y Saneamiento* (JCAS); in Coahuila, the *Comisión Estatal de Aguas y Saneamiento* (CEAS); in Durango, the *Comisión de Agua del Estado de Durango* (CAED); in Nuevo León, the *Servicios de Agua y Drenaje de Monterrey* (SADM); and in Tamaulipas, the *Comisión Estatal del Agua en Tamaulipas* (CEAT).

#### State and inter-state multi-stakeholder platforms

The geodatabase includes five platforms:

- The Rio Grande Compact Commission — U.S. inter-state platform — monitors water flows and administers water-sharing among the states of Colorado, New Mexico, and Texas. We mapped its domain through three spatial layers: one representing the spatial scope of the Interstate Compacts (including the Rio Grande Compact), one identifying the gauges used to monitor and account for states’ water delivery obligations to each other, and one identifying the “post-Compact” reservoirs used to manage states’ water delivery obligations.
- The San Juan Chama Project (SJCP) — U.S. inter-state platform — moves water through a trans-mountain diversion from the Colorado River basin in the state of Colorado (San Juan River) to municipal and irrigation users in the upper and middle Rio Grande valley of New Mexico. We mapped this project with three vector datasets: one for the trans-mountain diversion, one for the reservoirs where imported water is stored and transferred, and one displaying the users of that water (counties, municipalities, irrigation districts, tribe).
- The Rio Grande Project — binational and inter-state platform — supplies water for irrigation from the Elephant Butte Reservoir to several U.S. and Mexican irrigation districts. Its domain is represented through two vector datasets: one representing the dams used for storage (Elephant Butte, Caballo) plus the diversion dams for distributing water in the U.S. and Mexico (Percha, Leasburg, Mesilla, American, Riverside — which is inactive, International); and one representing the irrigation districts supplied by the Rio Grande Project (EBID, EPWID#1 and DR 009 Valle de Juarez).
- The *Consejo de Cuenca del Río Bravo* (Rio Bravo Watershed Council) — Mexican inter-state platform — is a multi-state entity that develops and implements a basin management plan for the CONAGUA Hydrological-Administrative region VI in Mexico. We mapped its domain using its administrative boundary.
- The Rio Grande Basin Roundtable in southern Colorado — intra-state platform — regroups various stakeholders such as counties, municipalities, and conservancy districts to identify consensual strategies that meet competing needs in the region, and to provide support and funding for basin projects^29^. The geodatabase maps the boundary of its domain of action.

#### Groundwater-focused institutions

This dataset maps the local entities and related infrastructures associated with the administration, management, monitoring and conservation of groundwater in the U.S. states of Colorado, New Mexico, and Texas.

- In Colorado, we mapped three levels of groundwater management: the Rio Grande Water Conservation District (RGWCD) that manages groundwater at the regional level (San Luis Valley); the Groundwater Management Subdistricts that manage groundwater at the local level (subdistrict); and the high capacity wells with augmentation/replacement plans. For the regional level, we provided the spatial domain of the RGWCD, the location of unconfined and confined wells that the RGWCD monitors, and the boundaries of the Closed Basin, an unconfined groundwater salvage project maintained by RGWCD that transfers water from the aquifer to the surface water basin.

- The state of New Mexico is divided into 39 declared groundwater basins where the OSE assumes jurisdiction over the appropriation and use of groundwater. We mapped the 18 basins overlapping the RGB.
- In Texas, we mapped the domains of the Groundwater Management Areas (GMA), the Groundwater Conservation Districts (GCD) in charge of the implementation of groundwater management plans within the GMA, and the Priority Groundwater Management Areas (PGMA) located in areas with critical groundwater problems.

In Mexico, except for the groundwater permits (Títulos y permisos de aguas nacionales) available in the section Water Use & Hydraulic Infrastructures > Water Rights/Permits, we did not find any comparable administrative or governance mechanism explicitly set up on a spatial basis to manage groundwater per se.

At the transboundary level, the U.S. and Mexico also established a binational agreement, the Transboundary Aquifer Assessment Program (TAAP), to strengthen collaborations among Mexican and U.S. institutions to jointly assess priority shared aquifers along the U.S.-Mexico border^30^. Two of the four transboundary priority aquifers underlay the RGB: Mesilla/Conejos-Médanos and Hueco Bolson.

#### Irrigation distribution organizations

This sub-category maps the boundaries of the organizations that divert and coordinate surface water delivery to irrigators, and ensure maintenance of the irrigation conveyance systems, in both the U.S. and Mexico. Our dataset provides the name and the area managed by the organizations, in addition to other specific information for each state and country. We sought to gather the spatial boundaries of three organizations: the irrigation districts (*Distritos de Riego* in Mexico), ditch companies, and community ditch associations, an umbrella term under which we include the *acequia* systems of Colorado and New Mexico, and *Unidades de Riego* (*Irrigation Units*) across the states of Mexico. However, spatial datasets of ditch companies and community ditch associations were unavailable for New Mexico and Mexico.

#### Land management

This dataset maps four types of land management: Certified Ejido & Communal Land, Public, Native American/Tribal, and Private/Not Reported. The dataset results from the compilation of two national datasets: the National Surface Management Agency Area Polygons released by the Bureau of Land Management (BLM) that maps state and federally owned lands as well as Native American tribal lands in the U.S., and the *Perimetrales de los núcleos agrarios certificados* produced by the *Registro Agrario Nacional* (RAN) that maps *ejido* and *comunidad* (communal) lands in Mexico. When compiling the datasets, we labelled all areas that were neither classified by the BLM nor the RAN as “Private/Not reported”.

#### Protected areas

This dataset maps the areas designated as protected by the International Union for Conservation of Nature (IUCN).

#### Border control

This sub-category maps three key components of border control along the international reach of the Rio Grande/Río Bravo: the U.S. Southwest Border Patrol Sectors associated with the migration apprehension statistics; the border crossing ports of entry; and vehicle and pedestrian fences/barriers. U.S. border control is very active and has important implications for the management of riverbank and riparian areas. However, this research to-date has not covered policies or practices by Mexican border-related agencies that affect water management or flow.

#### Soil and water conservation districts (included only for the U.S.)

The vector dataset provided in this sub-category map the outlines of the Soil and Water Conservation Districts (SCWDs) in Colorado, New Mexico, and Texas. SCWDs foster voluntary conservation practices among private and public landowners, helping to manage and protect soil, water, forests, and wildlife at the local level. The SWCDs are a specific kind of district, with a mandate that is particular to the legal and institutional history of land and water management in the U.S. Our research to-date has not found anything equivalent to this formation in Mexico, and even less so, any spatial datasets for it.

#### Collaborative conservation projects

This sub-category includes conservation projects where members of multiple sectors and/or institutions actively work in collaboration to identify, protect, manage, monitor, and/or develop policy for the conservation of specific sites. Our research to-date has not extended to documentation of all such projects in either the U.S. or Mexico, and did not uncover spatial data sets that document their location. Our datasets map three collaborative conservation projects. Two of them, the Landscape Conservation Cooperatives (LCCs) and the North America Bird Conservation Joint Ventures (BJVs), are transboundary. For both, we mapped the boundaries of the four LCCs and the five BJVs that span the RGB. While the U.S. Fish and Wildlife Service recently discontinued its funding for the LCCs network, we decided to include these cooperatives in our dataset since the LCCs generated highly relevant information for the RGB in the past. The third project refers to the Instream Flow Program in Colorado. For this project, we mapped the streams and lakes affected by the Instream Flow Program.

### Hydrology (HYD)

The Hydrology category includes five surface water and groundwater related sub-categories.

#### Watershed boundary

The geodatabase provides the outlines of the Rio Grande/Río Bravo basin and of its sub-basins. The watershed boundary dataset is derived from the World Wildlife Fund’s (WWF) HydroSHEDS product, created from NASA’s Shuttle Radar Topographic Mission (SRTM) 15-second Digital Elevation Model (DEM) and released by the Food and Agriculture Organization (FAO) geoportal.

#### River networks

This dataset of vectorized river reaches, derived from the WWF HydroSHEDS products, was also accessed from the FAO geoportal.

#### Aquifer boundaries

We mapped the aquifer boundaries by assembling two national geospatial datasets compiled by the National System of Water Information of CONAGUA and the USGS.

#### Gauging stations

The geodatabase provides datasets for the location of discharge monitoring stations operated by USGS (above Elephant Butte Dam), IBWC/CILA (below Elephant Butte Dam and along the border to the Gulf of Mexico), and CONAGUA (tributaries of the Rio Grande/Río Bravo coming from Mexico). Each dataset provides a URL to the historical time series of the stream flow.

#### Water quality stations

This dataset provides the location of water quality stations monitored by IBWC and TCEQ for the U.S. portion of the RGB.

### Water Use & Hydraulic Infrastructures (USE)

Water Use & Hydraulic Infrastructures datasets provide information related to consumption and diversion of water.

#### Withdrawals

This dataset provides estimates in million cubic meters per year of water used at the U.S. county and MX *municipio* level. For both countries, estimates are available by type of source (surface or groundwater) and by sector of use (e.g., public supply, domestic, industrial, irrigation, thermoelectricity). For the U.S., the data set also breaks down estimates by fresh water and saline water.

#### Irrigation

We provide two datasets (one per country), which reports on a county/*municipio* basis the irrigated area estimated in hectares and the spatial coverage of different systems of irrigation (surface, sprinkler, micro-irrigation and other), estimated in hectares for U.S. and in number of farms for Mexico. For the U.S., we used the original classes (surface, sprinkler, micro-irrigation) and for Mexico, we clustered as follows: lined and earthen canals in surface; sprinkler and micro-sprinkler in sprinkler; and drip in micro-irrigation.

#### Water rights/permits

This sub-category displays the allocation of surface water rights and groundwater rights at the state level for U.S. and Mexico. A water right is a property right that conveys the right to use a certain amount of water. These are administered by the Offices of the State Engineer in the U.S. and by CONAGUA in Mexico. The datasets report the geographic coordinates of the water right holder, and when available, the amount of water right authorized (in acre feet in the U.S. and in cubic meters in Mexico), the name of the water right holder, the adjudication date, the rank of priority (i.e., the water right’s seniority in a water drainage), or the type of use.

#### Dams

We provide two vector datasets. The first one, derived from the National Inventory of Dams, maps all dams in the U.S. exceeding a certain height and capacity of storage. The second one maps all dams recorded by the CENAPRED in Mexico.

#### Water diversion structures

The geodatabase provides three vector datasets of water diversion structures (ditch/canal, pipeline, underground conduit and connector). The first one covers the five MX states and is derived from a Mexican national data source. The second one covers the three U.S. states and is derived from a U.S. national data source. The third one covers Colorado with more details and is derived from a state data source.

#### Wells (included only for the U.S.)

We compiled four datasets of well locations. One is derived from the National Hydrography Dataset (NHD) for the whole U.S. portion of the RGB; the three other ones were compiled by the OSE for each U.S. state and provides more details. Our research to-date has not uncovered data for wells in Mexico.

### Socio-Economics (SE)

This category includes five datasets related to demography, economics, and transportation networks.

#### Population

This vector dataset reports the total population for the years 1990, 2000, and 2010 on a county/*municipio* basis. We assembled national demographic census information distributed by the U.S. Census Bureau and INEGI.

#### Population density

This raster dataset depicts population density (number of persons per square kilometre) at 30 arc-second resolution (approximately 1 km at the Equator) for the years 2000, 2005, 2010, 2015, and 2020.

#### Income

Due to the lack of consistent data for both countries, the spatial database includes two heterogeneous datasets: the personal income for the U.S. counties from 1969 to 2015 and the income distribution (i.e., the distribution of the working population per category of income) in 1990, 2000, and 2010 for the Mexican *municipios*.

#### Number and size of farms

This sub-category provides county/*municipio*-based statistics about the number of farms, the total farmed area, and the average farm size (in 2007 and 2012 for the U.S. and in 2007 for Mexico).

#### Transport infrastructures

This sub-category contains two datasets: the roads derived from the Global Roads Open Access Data Set, Version 1 (gROADSv1) and the railroads derived from the North American Environmental Atlas.

### Biophysical environment (BIO)

The biophysical environment category provides ecological, pedological, topographical, and land-use/land-cover datasets.

#### Ecoregions

Ecoregions are areas with similar patterns of biotic and abiotic phenomena, including geology, physiography, vegetation, climate, soils, land use, wildlife, and hydrology. Ecoregions can be mapped for different nested levels. For North America (Canada, U.S. and Mexico), data are available for three levels (Level I being the coarsest and Level III the finest). Ecoregions level IV have also been delineated for the U.S. but not yet for Mexico. Each of those datasets are included in the geodatabase, although ecoregion Level IV is missing for Mexico.

#### Habitat

We compiled six national datasets due to a lack of transboundary coverage. In the U.S., we mapped wetlands and deepwater habitats (extent, approximate location, and type), riparian areas, and critical habitat for endangered and threatened species. In Mexico, we mapped Ramsar wetlands sites (representative, rare or unique wetlands that are important to conserve biological diversity), priority sites for restoration (areas of high biological value that require restoration actions), and priority focus sites for biodiversity conservation (conserved habitats that host endangered and threatened species and are adjacent to the protected areas).

#### Soil cover

This vector dataset displays different soil units based on the FAO taxonomy at 1:5,000,000 scale.

#### Elevation

This raster data, derived from the Harmonized World Soil Database (version 1.1), provides the median elevation aggregated to 30 arc-second grid cells. It is derived from the NASA Shuttle Radar Topographic Mission Digital Elevation Model and the USGS GTOPO30.

#### Slope

We calculated the median slope in percent and in degree at a 30 arc-second resolution from the elevation data described above, by using the *Spatial Analyst* tool Slope in ArcGIS.

#### Land cover

This raster dataset, derived from the North American Land Change Monitoring System (NALCMS), depicts the land cover in the RGB, based on 2010 Landsat satellite imagery. NALCMS uses the level II of the Land Cover Classification System (LCCS) standard developed by FAO.

#### Land use

Due to a lack of consistent transboundary datasets, we gathered national datasets to map land-use in the RGB. For the U.S., we used the yearly USDA Cropland Data Layer, a high-resolution crop-specific land-use image, available from 2008 to 2018 at a 30-meter resolution. For Mexico, we derived our datasets from two governmental datasets. The first one is a spatial dataset of land uses for 2011 and 2014 at a 30-meter resolution, with a coarse classification of crops (rain-fed *vs* irrigated crops). The second one, non-spatial, is derived from the 2007 Census and provides on a *municipio* basis planted area estimates (in hectares) of annual spring and winter crops and perennial crops.

### Data gaps

Although we developed an extensive database, it is not comprehensive for either the U.S. or Mexico. Our approach led us to identify several spatial data gaps for socio-environmental research in the RGB.

#### Yearly crop-specific datasets for Mexico

Supplying yearly crop-specific time-series datasets at a 30-m resolution will be helpful for comparing cropland dynamics in the U.S. and Mexico.

#### Boundaries of community ditch and acequia associations (in NM) and Unidades de Riego (MX)

To the authors’ knowledge, these spatial boundaries are not publicly available. Mapping them is important, because of their water management implications. The NMOSE is currently working on updating and assembling this information for New Mexico^31^.

#### Land ownership

For Mexico, we did not find any spatial datasets delineating public lands, except the Certified Ejidos and Communal Lands. Generating a dataset that differentiates, in more detail, public from private lands in Mexico would be valuable for land and water management research.

#### Groundwater wells (MX)

We were not able to access a spatial dataset of wells for Mexico. Mapping well locations is of primary interest for surface and groundwater management in the basin.

#### Binational infrastructures

Some information on binational infrastructure is missing, such as the Morillo drain (described in Supplementary Information 1).

Climate records are also critical for understanding hydrological processes in the basin. While not included in our geodatabase, Livneh and others^11^ published a transboundary historic dataset that derives observed daily precipitation, minimum and maximum temperature gridded to a 1/16° (~6 km) resolution for the time period 1950–2013.

## Technical Validation

To ensure the reliability of the spatial database, we draw on a critical assessment of three factors: the quality of the source of information, transboundary discontinuities, and geoprocessing.

Regarding the sources of information, we sought to download most of the raw datasets from official sources such as intergovernmental organizations and governmental agencies (e.g., FAO, USIBWC, USGS, USDA, CONAGUA, INEGI, CONABIO, CONANP, CODWR, NMOSE, TCEQ) or from joint projects overseen by reputable universities, research institutes, and non-governmental organizations (e.g., from the NASA Socioeconomic Data and Applications Center, the North American Environmental Atlas). We compiled a few datasets (around 6%) from private entities (associations, districts) as they were the only source of the desired information. For example, this is the case for the border fences. We downloaded the spatial data from Reveal from The Center for Investigative Reporting and OpenStreetMap contributors (https://github.com/cirlabs/border_fence_map) who traced the fences along the border by using open-source mapping tools and by digitizing detailed PDF maps obtained through a Freedom of Information Act request^32^. The creators of the data set acknowledge slight differences with the official government agency numbers. However, this is the most detailed border fence map publicly available. We also gathered four datasets — that were not downloadable online — by email requests from three organizations. The RGWCD provided the boundaries of the RGWCD and the Groundwater Management Subdistricts #1, #2, #3, #4, #5, #6 in Colorado. The CSCB (Colorado State Conservation Board) and the TSSWCB (Texas State Soil and Water Conservation Board) shared the boundaries of the SWCDs of Colorado and Texas, respectively. Although these datasets were not gathered from governmental sources, we believe that these datasets are the best available and we rely on the accuracy of data collection of the three organizations.

Whenever possible, we used global or bi-national input datasets to avoid inconsistencies related to differences in resolution, type of information, or geoprocessing methods among counties and states. Around 11.5% of our raw datasets have a global or bi-national spatial extent, and therefore cross international and state boundaries. Nevertheless, a significant amount of the raw datasets we gathered were nation-wide (70%) or state-wide (18.5%), which could lead to some of the inconsistencies previously mentioned. All information gathered from Mexican sources was translated from Spanish to English and verified by a bilingual speaker.

Regarding the geoprocessing operations, for all datasets, we used several tools and functions available on ArcGIS, such as Project, Clip, Dissolve, Slope, Join, Merge and we rely on the accuracy of this software to create the final outputs. A series of quality checks has been carried out to catch as many potential coding errors as possible. Attributes were checked by using visual inspection. For quality control, transparency, and reproducibility, processing steps are thoroughly detailed in the metadata of each dataset.

## Usage Notes

This spatial database offers a wide range of applications for transboundary and interdisciplinary research. We provide three examples drawing from our own research.

**Fig. 2:**
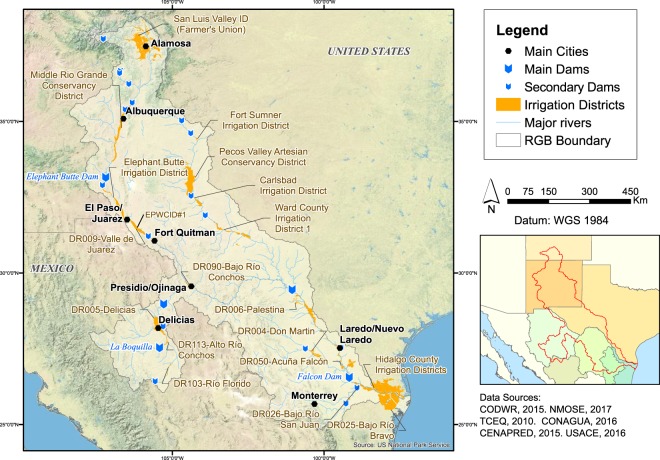
Map of the main dams and irrigation districts in the Rio Grande/Río Bravo basin.

**Fig. 3:**
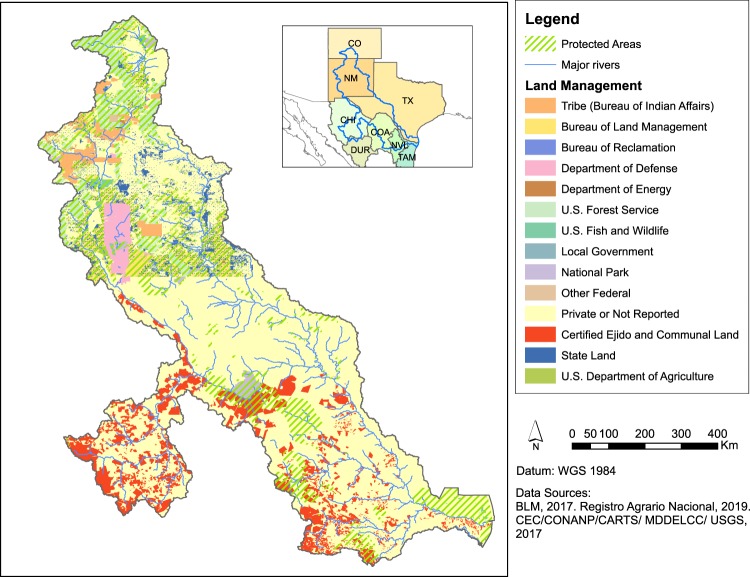
Map of the Rio Grande/Río Bravo basin depicting land ownership, protected areas, and major rivers, generated from the geospatial datasets of the geodatabase.

1. A broad range of mapping products can be created for the whole RGB or sub-sections, such as a map of the main irrigation districts and dams in the RGB (Fig. 2), a map of land managers and protected areas (Fig. 3), and a comparative map of water use and population on a county/*municipio* basis (Fig. 4).

**Fig. 4:**
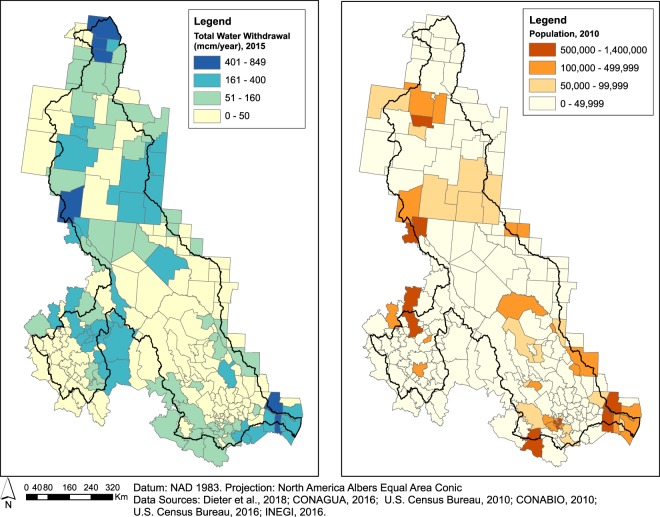
Map comparing the total amount of water withdrawals (millions of cubic meters per year) in 2015 with the 2010 population on a county/*municipio* basis.
2. The geodatabase can support spatially explicit and integrated modelling for the RGB^27^. We are currently developing a simulation model for the RGB that draws on water and land governance datasets (political jurisdiction boundaries, land management and protected areas, irrigation district outlines), as well as hydrologic and biophysical environment datasets (river network, elevation, water bodies, gauges, land-use), socio-economic (population), and water use and hydraulic infrastructures (dams).
3. The geodatabase can be used to conduct spatiotemporal and geostatistical analyses. Examples of interdisciplinary topics include: (i) evaluating whether water use correlates with environmental variables (ecoregions, land use), socio-economic factors (population), or governance (e.g., related to land ownership, irrigation district, or state); and (ii) assessing land-use change at multiple scales (local, state, national). For example, we are currently analysing the spatial patterns of land fallowing in the basin.

Users of the compiled datasets should cite this data paper following the recommended citation format of the journal. Furthermore, we encourage users to refer to the sections “Credits” and “Use limitations” in the metadata of each layer file, as additional data constraints and required credits of the original data sources may apply.

In conclusion, to achieve more sustainable transboundary water management, it is necessary to approach problems from a boundary-spanning and interdisciplinary perspective. By creating a basin-wide, socio-environmental geodatabase for the RGB, we address this issue and support further interdisciplinary research in the basin. The main novelty of our approach was to include geospatial datasets mapping public and private water governance, which contributes to better understanding of scale interactions and mismatches between ecological and social (decision-making) boundaries^33^. Moreover, we were able to identify data gaps, especially for the Mexican side of the RGB, and therefore the need for further research to reduce data imbalance between the two sides of this transboundary basin. Even though our study is limited to the socio-environmental boundaries of the RGB system and the typology of actors is specific to the shared basins between U.S. and Mexico, we consider that our approach to assembling a wide range of data is transferable to other shared river basins to serve transboundary and cross-disciplinary collaboration and research.

### Supplementary information

Supplementary Information 1

Supplementary Information 2

Supplementary Information 3

## Data Availability

We created the individual output datasets using geoprocessing scripts in the Python programming language and the arcpy package. All geoprocessing scripts are available on GitHub and are accessible through the Open Science Framework (OSF) repository^28^.

## Online-only Table

**Online-only Table 1:** List of geospatial layers for *Water & Land Governance* including their description, format and input data sources.

Datasets	Output geospatial layers	Description	Format	Input source
Political Jurisdication Boundaries	1.Nations 2.States 3.Counties_and_municipios 4.Places 5.Populated_Places	1.U.S. & MX boundaries 2.State boundaries 3.RGB counties and municipios 4.RGB places (cities, towns, villages) 5.RGB populated places	point, polygon	U.S. Census Bureau^76^, INEGI^77^, INEGI/NRCan/USGS/CEC^78^
Binational Water Management Agencies	Binational_Dams	Reservoirs and diversion dams	point	USACE^47^
	IBWC_Offices	IBWC offices location	point	USIBWC^39^
	Canals Culverts Drainage_ditches Gates^a^ Laterals^b^ Levees Mow_Areas Ramps Ripraps ToeDrains^c^ Wasteways^d^	Levee Infrastructure (US)	point, line, polygon	
Federal water management agencies	1.US_Federal_Dams 2.MX_Federal_Dams	1.U.S. federally owned dams 2.Dams operated by CONAGUA	point	USACE^47^, CENAPRED^48^
State and intra-state water management agencies	*Colorado* 1.CDWR_div3 2.CDWR_div3_districts	Boundaries of: 1.Colorado Division 3 of Water Resources 2.Water Districts in Division 3	polygon	CWCB/DWR^51^
	*New Mexico* 1.NMOSE_WaterMaster_RG 2.NM_WaterPlanningRegions_RG	Boundaries of: 1.NMOSE Water Master Districts 2.Regional Water Planning areas	polygon	NMOSE^44^
	*Texas* 1.TCEQ_service_regions_RG 2.TWDB_RWPA_RG 3.TWDB_Regional_Project_Team_RG 4.TWDB_IFSS_Offices_RG	Boundaries of: 1.TCEQ regions 2.TWDB Water Planning Regions 3.TWDB Regional Project Teams 4.Inspection & Field Support Services Offices	polygon	TCEQ^79^ TWDB^52^
	*Mexico* 1.MX_State_Conagua_Offices 2.JCAS_Chihuahua 3.CEAS_Coahuila 4.CAED_Durango 5.SADM_Nuevo_León 6.CEAT_Tamaulipas	Boundaries of: 1.States Conagua offices 2.Junta Central de Agua y Saneamiento in Chihuahua 2.Comisión Estatal de Aguas y Saneamiento de Coahuila 3.Comisión de Agua del Estado de Durango 4.Servicios de Agua y Drenaje de Monterrey 5. Comisión Estatal del Agua de Tamaulipas	polygon	INEGI^77^
State and Inter-State Multi-Stakeholder Platforms	*Rio Grande Compact Commission* 1.Interstate_Compacts 2.RG_Compact_Gauges 3.RG_PostCompact_Dams	1.Interstate Compacts geographic domain 2.Gauges for accounting for states’ water obligations 3.”Post-Compact” dams for holdover storage of water for respecting states’ water obligations	polygon	USGS/USDA-NRCS^80^, USGS^38^, USACE^47^
	*San Juan Chama Project-SJCP* 1.SJCP_Tunnel 2.SJCP_Reservoirs 3.SJCP_Users	1.Transmountain diversion from the Colorado to the Rio Grande basin 2.Reservoirs used for importation and storage 3. Users of the SJCP water (irrigation districts, counties, municipalities, tribes, recreation)	line, polygon	USGS-NHD^49^, NMOSE^44^, U.S. Census Bureau^76^
State and Inter-State Multi-Stakeholder Platforms	*Rio Grande Project* 1.RG_Project_Dams 2.RG_Project_IrrigDistricts	1.Dams used for storage and diversion of water under the RG project 2.Irrigation districts supplied by the RG Project	polygon	USACE^47^, NMOSE^44^, TCEQ^79^, CONAGUA^37^
	*Consejo de Cuenca - Río Bravo* MX_Consejo_Cuenca	Hydrological-Administrative region (VI) of the Río Bravo	polygon	CONAGUA^81^
	*Rio Grande Roundtable* CO_RG_Roundtable	Rio Grande Roundtable in Colorado	polygon	U.S. Census Bureau^76^
Groundwater Focused Institutions	*Colorado* 1.CO_RGWCD_bound 2.CO_ClosedBasin_bound 3.CO_RGWCD_Wells 4.CO_Subdistricts_bound 5.CO_Augment_replacement_plans	1.Spatial scope of Rio Grande Water Conservation District 2.Boundaries of the Closed Basin 3.Groundwater Management Subdistricts boundaries 4.Unconfined and confined wells monitored by RGWCD 5. Augmentation/replacement plans	polygon, points	CDWR^43^, RGWCD^82^, RGWCD (shared)
	*New Mexico* NM_declared_GW_basins	Declared groundwater basins	polygon	NMOSE^44^
	*Texas* 1.TX_GMA 2.TX_GCD 3.TX_PMGA	1.Groundwater Management Areas 2.Groundwater Conservation Districts 3.Priority Groundwater Management Areas	polygon	TCEQ^79^, TWDB^52^
	*Transboundary* TAAP_Aquifers	Mesilla Basin/Conejos Médanos and Hueco Bolson aquifers	polygon	Teeple (2017)^83^, Driscoll & Sherson (2016)^84^
Irrigation Distribution Organizations	1.CO_Irrigation_Organizations 2.NM_Irrigation_Districts 3.TX_Water_Districts 4.MX_Distritos_Riego	1.Irrigation districts, ditch companies, community ditch associations in Colorado 2.Irrigation districts in New Mexico 3.Water districts in Texas 4.Irrigation districts in Mexico	polygon	CWCB/DWR^51^, NMOSE^44^, TCEQ^79^, CONAGUA^37^
Land Management	Land_Management	Boundaries of public, Native American, communal and private land in U.S. and Mexico	polygon	BLM^85^, RAN^86^
Protected Areas	ProtectedAreas	Geographic scope of protected areas in U.S. and Mexico	polygon	CEC/CONANP/CARTS/ MDDELCC/ USGS^87^
Border Control	1.Border_Crossings 2.Border_Fences 3.Border_ Sectors	1.U.S.-Mexico Border crossing ports of entry 2.Vehicle and pedestrian fences along the U.S.-Mexico border 3.Southwest Border Patrol Sectors and Migration Apprehension Statistics	point, line, polygon	HIFLD^88^, The Center for Investigative Reporting (https://github.com/cirlabs/border_fence_map), HSIP^89^, USCBP^90^
Soil and Water Conservation Districts	SWCD	Soil and Water Conservation Districts in Colorado, New Mexico and Texas	polygon	CSCB (shared), NMOSE^44^, TSSWCB (shared)
Collaborative conservation projects	*Transboundary* 1.LCC_network 2.Bird_Conservation_JoinVentures	1.Landscape Conservation Cooperative Boundaries 2.Bird Conservation Joint Ventures	polygon	USFWS^91,92^
	*Colorado* 1.CO_ISF_Reaches 2.CO_ISF_Termini 3.CO_Lakes	1.Instream Flow reaches in Colorado 2.Instream Flow termini 3.Natural lake levels	point, line,	CWCB/DWR^51^

^a^These structures are used to open or close culverts from the top of the levee and prevent flood. ^b^Canals following along the right of way of another stream. ^c^Drains carry seepage water away from a dam or levee. d: Channels which contain discharged water from behind a levee.
