# Supplementary material for: A socio-environmental geodatabase for integrative research in the transboundary Rio Grande/Río Bravo basin
Source: Sci Data. 2020 Mar 6;7:80. doi: 10.1038/s41597-020-0410-1 (PMC7060182; doi:10.1038/s41597-020-0410-1)
Supplement: Supplementary file 1 — Supplementary Information 1 [file 41597_2020_410_MOESM1_ESM.docx]

### Supplementary Information 1: Typology of Water Management Actors Included in the Geodatabase

The following description of actors and the organization of the spatial water-land management typology is derived from the ethnographic research conducted by co-authors Paladino and Friedman between 2015 and 2018 in ten sections of the basin^1^, complemented by a literature review. A summary of the main actors included in the geodatabase is available in Figure 1.

1. ***Political jurisdictions***: The geodatabase includes four political jurisdiction divisions: nation, state, county/*municipio,* and cities/incorporated towns (places). In the U.S. portion of the RGB, states are subdivided into counties, a political jurisdiction that includes both urban (cities and towns) and rural areas. In Mexico, the *municipio* is a comparable, sub-state governmental jurisdiction also comprised of urban and rural areas. Some county and *municipio* governments in the RGB play a role in supplying domestic and industrial water to residents, and may be involved in water quality monitoring. Larger U.S. and Mexican cities in the RGB typically provide water supply, drainage, wastewater treatment, and water quality control for residents and industries within or adjacent to their boundaries. Smaller urbanized areas (e.g., incorporated and unincorporated towns in the U.S., *ejidos, comunidades, rancherías, colonias and colonias agrícolas* in Mexico) employ a variety of multi- or individual-household water supply, drainage, and wastewater approaches.

The geodatabase also provides a point dataset of the most populated places in both countries.

2. ***Binational Surface Water Management Agencies***: Mexico’s *Comisión Internacional de Límites y Aguas* (CILA) and its US counterpart, the International Boundary and Water Commission (IBWC), conduct joint missions regarding boundary demarcation, allocation of bi-national waters; maintenance of river channels, habitat, levees, and infrastructure; and management of water quality sanitation, and flood control in the border region. Regarding water allocation, CILA and IBWC oversee the implementation of the U.S.-Mexico treaties of 1906 and 1944 (and others) for the RGB from south of Elephant Butte Dam to the Gulf of Mexico. Under those agreements, they monitor water flow and storage; negotiate the quantity, timing, and bi-national distribution of reservoir releases; operate international dams and reservoirs (Amistad and Falcon reservoirs, diversion structures such as American Dam, International Dam, Anzalduas Dam, and Retamal Dam). Regarding joint missions on flood control, IBWC and CILA operate and maintain three systems: the Upper Rio Grande Flood Control System, the Presidio Valley Flood Control System and the Lower Rio Grande Flood Control System^2^.

3. ***Federal* *surface* *water management agencies***: Both in the U.S. and Mexico RGB, federal agencies support inter-state water-sharing agreements, federal dam and reservoir management, flow and water quality accounting and monitoring. In the U.S. portion, the missions are distributed among several Federal agencies, including the Bureau of Reclamation (USBR), the U.S. Army Corps of Engineers (USACE), and the U.S. Geological Survey (USGS). In the Mexico portion, those responsibilities are centralized in the *Comisión Nacional del Agua* (CONAGUA). CONAGUA also administers water rights.

4. ***State and intrastate surface* *water management agency***: State and intra-state water management agencies exist in both the U.S. and Mexico RGB.

In the Mexico RGB, there are state-level offices of the federal agency CONAGUA. Each state also has entities that engage in planning, coordination, and oversight of domestic water supply and wastewater treatment state-wide, but can have wider scopes of action related to surface water use. We include here: in Chihuahua, the *Junta Central de Agua y Saneamiento* (JCAS); in Coahuila, the *Comisión Estatal de Aguas y Saneamiento* (CEAS); in Durango, the *Comisión de Agua del Estado de Durango* (CAED); in Nuevo León, the *Servicios de Agua y Drenaje de Monterrey* (SADM); and in Tamaulipas, the *Comisión Estatal del Agua en Tamaulipas* (CEAT)^3^.

In the U.S. RGB, State Engineers and their agencies administer water rights and interstate compacts. The Offices of the State Engineer and respective agencies also decentralize many water management functions to regional offices defined by watershed or political jurisdiction boundaries.

- In Colorado, the Office of the State Engineer is in the Colorado Division of Water Resources (CDWR). Its Division 3 is in charge of the RGB and allocates water in order of priority, monitors stream flows to assure delivery of RG Compact obligations (described hereafter), and establishes how much can be distributed to surface water rights holders. Division 3 is divided into eight districts.
- In New Mexico, surface water administration is under the responsibility of the New Mexico Office of State Engineer/Interstate Stream Commission (NMOSE-ISC). The state is divided into seven NM Water Master districts, four of which manage surface waters of the RGB (I-Albuquerque, II-Roswell, IV-Las Cruces and VI-Santa Fe). Each of these districts is staffed by one or more Water Masters, assigned to sub-basins of the RGB, who administer the distribution of water from the stream on a day-to-day basis. The state is also divided into Water Planning Regions, fourteen of which cross the RGB.
- In Texas, two institutions manage surface water of the Rio Grande. First, the Texas Commission on Environmental Quality (TCEQ) administers water rights and interstate compacts as well as water quality and watershed protection. The state is split into sixteen TCEQ service regions, six of which cover the RGB. Second, the Texas Water Development Board (TWDB) coordinates state water planning, provides technical assistance and funding, and approves groundwater management plans. The state is divided into sixteen Water Planning Regions, five of which overlap the RGB. TWDB also delineates geographic areas for Regional Project Teams (three in the RGB) and Inspection & Field Support Services Offices (two in the RGB). A third actor is the Rio Grande Watermaster that monitors, schedules diversions, and allocates water among diverters from Fort Quitman to the Gulf of Mexico, including a portion outside of the watershed - the Nueces-Rio Grande Costal Basin lying in Hidalgo, Cameron, Willacy, and Starr Counties. Below Amistad reservoir, surface water is administered via the Watermaster in conjunction with the Amistad-Falcon reservoir system. The Pecos & Devils River watersheds, Bolson Closed Basin, and the Upper Rio Grande north of Fort Quitman are non-Watermaster areas. However, the outlines of the Watermaster & non-Watermaster areas are not released in the public domain, and therefore not included in the geodatabase.

5. ***State and*** ***Inter-State Multi-Stakeholders Platforms****:* This sub-category describes multi-stakeholder platforms, representing actors from public and private water institutions that make water management decisions at a sub-basin or multi-state level. We include five in the RGB: the Rio Grande Compact Commission (in U.S.), the San Juan Chama Project (between Colorado and New Mexico), the Rio Grande Project (between U.S. and Mexico), the *Consejo de Cuenca del Río Bravo* (Rio Bravo Watershed Council in Mexico) and the Rio Grande Basin Roundtable (southern Colorado).

- The **Rio Grande Interstate Compact Commission** — US inter-state platform — monitors water flows and administers water sharing among the states of Colorado, New Mexico, and Texas as agreed upon in the 1938 Rio Grande Compact. It includes Engineer Advisors from those three states, plus a federal representative. Engineer Advisors use a set of gauges and “post-Compact” reservoirs for accounting and fulfilling states’ water delivery obligations. The “post-Compact” reservoirs correspond to all reservoirs constructed after 1937 above Lobatos (CO) and after 1929 above San Marcial (NM), where the upstream state must always hold an amount of water equal to the extent of its accrued debt to the downstream state, within the physical limitations of their storage capacity. Two other Interstate Compacts Commissions administer water sharing of two other rivers in the RGB: the Costilla Creek Compact, a joint agreement between the states of Colorado and New Mexico for the use of the waters of the Costilla Creek and the Pecos Compact, a joint agreement between the states of New Mexico and Texas for the use of the waters of the Pecos river.
- The **San Juan Chama Project** (SJCP) — US inter-state platform — moves water through a transmountain diversion from the Colorado River basin (San Juan River in Colorado) to the northern and middle Rio Grande sections of New Mexico. Imported water is diverted through a series of three tunnels (Blanco, Oso, and Azotea) and stored in Heron Reservoir located on Willow Creek near the confluence with the Rio Chama. SJCP’s water are delivered for irrigation, municipal, domestic, industrial, recreation, and fish and wildlife purposes^3^. The main contractors are: the City of Albuquerque (48,200 ac-ft), the Middle Rio Grande Conservancy District (20,900 ac-ft), Jicarilla Apache Tribe (6,500 ac-ft), the City and County of Santa Fe (5,605 ac-ft), Cochiti Reservoir Recreation Pool (5,000 ac-ft), Los Alamos County (1,200 ac-ft), Pojoaque Valley Irrigation District (1,030 ac-ft) and other small municipalities such as Española, Belen or Taos.
- The **Rio Grande Project** — binational and inter-state platform — provides water for irrigation from Elephant Butte Reservoir to south-central New Mexico and west Texas, as well as to Juarez Valley (in the state of Chihuahua) under the terms of the Convention of 1906. Two dams are used for storage (Elephant Butte Dam and Caballo Reservoirs) and six for diversion of water from U.S. to Mexico (Percha, Leasburg, Mesilla, American, Riverside – now inactive, and International). Elephant Butte Dam also generates hydroelectric power for communities and industries in southern New Mexico. Water release and distribution decisions are made by a committee of IBWC/CILA, USBR, and three irrigation districts (EBID in New Mexico, EPWID#1 in Texas, and DR 009 Valle de Juarez in Chihuahua) according to water supply projections (estimated by USBR) and water demand.
- The ***Consejo de Cuenca del Río Bravo*** (Rio Bravo Watershed Council) — Mexican inter-state platform — brings together representatives of federal, state, and municipal agencies, as well as water user sectors and civil society organizations to collaboratively develop and implement a watershed management plan for the CONAGUA Hydrological-Administrative region (VI) of the Río Bravo. It is also involved in building basin-wide data sets and modelling. Its scale of action is wider than the catchment boundary of the Río Bravo^4^.
- The **Rio Grande Basin Roundtable** — intra-state platform — in southern Colorado regroups counties, municipalities, conservancy and conservation districts and other entities (*acequias*, well owners, etc.) to define projects and methods that meet municipal, industrial, agricultural, environmental and recreational water needs in the region. Among other actions, the Rio Grande Basin Roundtable has provided technical and financial support to the Rio Grande Basin Implementation Plan.

6. ***Groundwater Focused Institutions***: In the U.S., multiple entities are involved in the administration, management, monitoring, and conservation of groundwater.

- In Colorado, we distinguish four levels:

- In addition to issuing water well permits, the Office of the State Engineer is in charge of preparing Well Rules and Regulations governing the use of underground water in Division 3. As a response to water table decline due to prolonged drought and increased groundwater consumption in the early 2000s, the Well Rules and Regulations aim to prevent injury to senior water rights holders, regulate use of the confined and unconfined aquifers, and prevent interference with Colorado’s obligations under the Rio Grande Compact^5^.

- The Rio Grande Water Conservation District (RGWCD), formed in 1967, is a corporate body and political subdivision that ensures protection, enhancement, and development of water resources in the San Luis Valley, a heavily groundwater-irrigated area. The district encompasses a five-county region (Alamosa, Rio Grande, Conejos and portions of Saguache and Mineral Counties), including the Closed Basin. The RGWCD monitors a set of more than 100 unconfined and confined wells to assess changes in aquifer storage over time. The RGWCD provides maintenance to the Closed Basin Project, an unconfined groundwater salvage project operated by the U.S. Bureau of Reclamation. The purpose of the project is to assist Colorado in meeting its commitment under the Rio Grande Compact, and to provide water to the Alamosa National Wildlife Refuge and Blanca Wildlife Habitat Area^6^. The RGWCD also provides assistance to the Groundwater Management Subdistricts described next.

- As a response to the Well Rules and Regulations, Groundwater Management Subdistricts have been created to provide locally-generated water management plans to meet state regulations^1^. Special Improvement District No. 1 was the first one to be formed in 2006 and, more recently, between 2016 and 2018, five other subdistricts have been created across the valley with the help of the RGWCD.

- High-capacity wells with augmentation/replacement plan: In line with the Well Rules and Regulations, the owners of high capacity wells have either to join a Groundwater Management Subdistrict, or implement an augmentation/replacement plan for their well, or cease the use of their well^5^.

- In New Mexico, the Office of the State Engineer assumes jurisdiction over the appropriation and use of groundwater from 39 declared groundwater basins, representing extensions of land that have groundwater sources with reasonably ascertainable boundaries.
- In Texas, the TWDB approves groundwater management plans that are developed and implemented at the local level by county-based Groundwater Conservation Districts (GCD) to conserve, preserve, protect, recharge, and prevent waste of groundwater. GCD’s are further grouped into larger, regional Groundwater Management Areas (GMA)^7^, with eight GMA overlapping the RGB. In areas with critical groundwater problems, including shortages or contamination, Priority Groundwater Management Areas (PGMA) have also been defined with specific policies, rules, and plans. Two PGMA exist in the RGB.

In Mexico, we did not find any comparable administrative or governance mechanism explicitly set up on a spatial basis to manage groundwater per se. Although CONAGUA is supposed to record and monitor groundwater withdrawals and the municipal systems that pump groundwater manage it, the ethnographic research in the Rio Grande/Rio Bravo showed that groundwater use was generally described as unmonitored and very difficult to regulate. Groundwater pumping was free for all, until 2013 when the national government tried to put a stop to unlimited groundwater extraction over the whole country. Since 2013, there have been studies of aquifers to develop some degree of regulation of groundwater extraction. The aim is to have all groundwater users registered in a national registry with the amount of their water restrictions established. If some studies have been developed for about 55% of the country, in the rest of the country, including the northern states, they have been resisted^8^.

At the transboundary level, the U.S. and Mexico also established a binational agreement, the Transboundary Aquifer Assessment Program (TAAP), to strengthen collaborations among Mexican and U.S. institutions to jointly assess priority shared aquifers along the U.S.-Mexico border. Two of the four priority aquifers underlay the RGB: Mesilla/Conejos-Médanos and Hueco Bolson. The objectives of the TAAP are to create shared databases on groundwater quantity and quality, assess the interaction with surface water, and analyse groundwater management institutions and policies^9^.

7. ***Irrigation Distribution Organizations***: In this category, we include organizations that divert surface water and coordinate its delivery to irrigators (amount and scheduling), and ensure maintenance of the conveyance systems. Our dataset groups the large variation in organizational types present in the RGB into three main categories, based on some general variables of legal status (governmental or quasi-governmental, non governmental, communal), the source of water supply (reservoir or direct stream flow), the kind of infrastructure, and labour patterns.

- Irrigation Districts in the U.S. and *Distritos de Riego* in Mexico are large organizations, governmental or quasi-governmental, typically with an employed workforce (e.g., engineers or consulting hydrologists) and financial and technical resources to invest in improved-efficiency technologies for water conveyance and irrigation. They are almost exclusively associated with reservoirs, distributed along the main RGB stream and biggest tributaries.
- Ditch Companies are relatively smaller, private, not-for-profit or for-profit organizations, typically with a small, employed workforce. They may or may not operate reservoirs, and may contract access to storage water with reservoir owners, which may be private or public. In the RGB, they are more common in the state of Colorado.
- Community Ditch Associations are small, not-for-profit organizations, typically made up of small-scale farmers, many with centuries-long ties to place. Individually-variable governance structures, based on internal agreements that are often not legally codified, tend to set the rules of water distribution and maintenance. In contrast with irrigation districts/*distritos de riego* and ditch companies, they more frequently rely on labour provided by irrigator members, and often do not have access to financial or technical resources for investing in the efficiency of water conveyance and irrigation systems. They generally do not have direct access to reservoir storage unless they are affiliated with an irrigation district. In this category, we have included both the *acequia* associations of the upper RGB (southern Colorado and New Mexico) and the *Unidades de Riego* (“Irrigation Units”) of Mexico, many of which (especially the *acequias*) have Spanish colonial and indigenous origins.

*8.* ***Land Management***: There is a great variety of land manager types in the RGB. We identified four main types:

- Public: Public lands include state and federally-owned lands. In the U.S. portion of the RGB, they are managed by the following agencies: the Bureau of Land Management (BLM), the Bureau of Reclamation (BOR), the Department of Defense (DOD), the Department of Energy (DOE), the U.S. Forest Services (USFS), the U.S. Fish and Wildlife Services (USFWS), the State and National Parks Services (SPS and NPS), the U.S. Department of Agriculture (USDA) and the Local Government.
- Native American/tribal: Most of Native American/tribal lands are federally-recognized, sovereign territories with individually-varying governance structures, and communal land tenure systems that combine collective and individual use rights. The U.S. portion of the RGB includes 25 Native American reservations and pueblos, located in Colorado, New Mexico, and west Texas.
- Certified Ejido & Communal: Certified Ejido & Communal Lands define the lands managed by the *ejido* and the *comunidad* in Mexico. The *ejido* and the *comunidad*, derived from 20^th^ century land reform laws, combine collective ownership and governance with individual use rights exclusive to *ejido* or *comunidad* members, though the land was technically held in trust by the Mexican government and registered in the *Registro Agrario Nacional* (RAN - National Agrarian Registry). The *comunidad* is typically associated with indigenous communities; ejidos may be indigenous, non-indigenous, or mixed^10^.
- Private and not reported: This corresponds to all areas designated as private or without other classification.

9. ***Protected Areas***: According to the International Union for the Conservation of Nature (IUCN), a protected area is an "area of land and/or sea especially dedicated to the protection and maintenance of biological diversity, and of natural and associated cultural resources, and managed through legal or other effective means." [IUCN's classification system](http://cms.iucn.org/theme/protected-areas/wcpa) distinguishes six groups^11^: Strict nature reserve/wilderness area (managed mainly for science or wilderness protection), National park (managed mainly for ecosystem protection and recreation), Natural monument (managed mainly for conservation of specific natural features), Habitat/species management area (managed mainly for conservation through management intervention), Protected landscape/seascape (managed mainly for landscape/seascape protection and recreation), Managed resource protected area (managed mainly for sustainable use of natural systems).

The RGB hosts a diversity of protected areas including the largest transboundary protected area of North America, comprised of two Mexican Flora and Fauna Protection Areas (Maderas del Carmen and Cañon de Santa Elena) and one U.S. National Park (Big Bend)^12^. Protected areas, especially in the Mexico portion of the RGB, may be owned by private landowners, with some degree of management by or management agreements with public agencies or non-governmental organizations, and may overlap the private or *ejido*/*comunidad* boundaries.

10. ***Border Control***: The Rio Grande/Río Bravo forms a natural border between U.S. and Mexico. Border control is very active and has important implications for the management of riverbank and riparian areas. For example, the U.S. [Immigration and Customs Enforcement](https://www.ice.gov/about) (ICE) ensures the cleaning of tall riparian vegetation on the riverbank (such as the giant cane and Tamarisk) to facilitate border patrol surveillance^1^. Our research to-date has not covered policies or practices by Mexican border-related agencies that affect water management or flow.

11. ***Soil and Water Conservation Districts (included only for the U.S.)***: Established under state legislature after the Dust Bowl in the 1930s, the Soil and Water Conservation Districts (SWCDs) are non-profit organizations spread out across the United States (typically drawn along county boundaries) that carry out natural resource management programs at the local level. The SCWDs’ role is mainly to foster voluntary conservation practices among private and public landowners to help manage and protect soil, water, forests, and wildlife^13^. Examples of conservation activities include operation and maintenance of flood control structures, rotational grazing, and wetland restoration. In the RGB, SWCDs are grouped in associations at the state level (Colorado Association of Conservation Districts^14^, New Mexico Association of Conservation Districts^15^, and the Association of Texas Soil and Water Conservation Districts^16^) and are represented by the National Association of Conservation Districts (NACD) at the federal level. SWCDs can receive technical and funding assistance from the U.S. Department of Agriculture - Natural Resources Conservation Service^17^ (USDA-NRCS) as well as from the State Department of Agriculture, such as the Colorado State Conservation Board^18^ and the New Mexico Department of Agriculture (NMDA^19^), or from other state agencies (such as the Texas State Soil and Water Conservation Board - TSSWCB)^20^.

The SWCDs are a specific kind of district, with a mandate that is particular to the legal and institutional history of land and water management in the U.S. Our research to-date has not found anything equivalent to this formation in Mexico, and even less so, any spatial data sets for it.

12. ***Collaborative conservation projects***: This category includes conservation projects where members of multiple sectors and/or institutions actively work in collaboration to identify, protect, manage, monitor, and/or develop policy for the conservation of specific sites. We included tree of them in our database. Two of them, the Landscape Conservation Cooperatives (LCCs) and the North America Bird Conservation Joint Ventures (JV), are transboundary. The other one acts at the state level.

- The Landscape Conservation Cooperatives were established in the early 2000s to provide science capacity and technical expertise for protecting natural and cultural resources across North America. The network brought together federal, state, and local governments with Native American tribes, non-governmental organizations, universities, and other public and private organizations to plan and act upon collaborative conservation issues^21^. Four LCCs were acting in the RGB: the Southern Rockies LCC, the Desert LCC, the Gulf Coast Prairie LCC and the Great Plains LCC. While the U.S. Fish and Wildlife Service recently discontinued its funding for the LCC network, we decided to include the cooperatives in our dataset since the LCCs generated highly relevant information for the RGB in the past.
- Migratory Bird Joint Ventures (BJVs) are cooperative, regional partnerships of agencies, organizations, corporations, tribes, or individuals that implement national or international bird conservation plans within a specific geographic area or for a specific taxonomic group. BJVs have expanded across all U.S. and Canada and much of Mexico since 1986^22^. The RGB is shared by five BJVs: Intermountain West, Playa Lakes, Rio Grande, Sonoran, and Gulf Coast.
- In Colorado, the Instream Flow Program, administered by the Colorado Water Conservation Board (CWCB), is a program for the appropriation, acquisition, protection, and monitoring of instream flow (ISF) and natural lake level water rights. The CWCB appropriates non-consumptive water rights for minimum flows on portions of the river and for minimum water levels in natural lakes. These rights are administered within the Colorado state’s water right priority system^23^.

Our research to-date has not extended to documentation of all such projects in either the U.S. or Mexico, and did not uncover spatial data sets that document their location. For example, we identified three other collaborative conservation projects that we did not represent in our geodatabase due to a lack of spatial data.

- The Rio Grande Headwaters Land Trust (RiGHT) is a non-profit local land trust in the San Luis Valley that work with private landowners, public agencies, and other conservation organizations to protect the private lands along the Rio Grande and its major tributaries. More than 10,000 hectares were protected as of 2015. RiGHT also provides estate planning, conservation easements, water issues, and sustainable agriculture educational programs^24^.
- The Rio Grande Headwaters Restoration Project is a partnership with federal, state and irrigation organizations in southern Colorado with several on-going restoration projects (e.g. the replacements of old and inefficient diversion and headgate, stream bank stabilization and riparian restoration)^25^.
- The Middle Rio Grande Endangered Species Collaborative Program is a multi-agency, multi-stakeholder initiative that was created to help ensure Endangered Species Act (ESA) compliance, and especially protect two endangered species along the Middle Rio Grande, the Southwestern willow flycatcher (*Empidonax traillii extimus*) and the Rio Grande silvery minnow (*Hybognathus amarus*)^26^.

**Figure 1**. Chart of water management actors represented in the geodatabase

### References

1 Koch, J., Friedman, J. R., Paladino, S., Plassin, S. & Spencer, K. Conceptual modeling for improved understanding of the Rio Grande/Río Bravo socio-environmental systems. *Socio-Environmental Systems Modelling.* **1** (2019).

2 U.S. International Boundary and Water Commissions (IBWC). *Lower Rio Grande Flood Control Project Texas Brochure*. El Paso, TX.

3 Fondo para la Comunicación y la Educación Ambiental A.C. *Directorio de Organismos Operadores de Agua Potable y Alcantarillado*. Agua.org.mx (2015).

4 Comisión Nacional del Agua (CONAGUA). *Estadísticas del Agua en México, edición 2015*. México, D.F. (2015).

5 DiNatale Water Consultants. *Rio Grande Basin Implementation Plan*. Alamosa, CO (Rio Grande Basin Roundtable, 2015).

6 Wolfe, D., Blaine, T., Gordon, P. R. & Simpson, H. *Calendar Year 2016 Report to the Rio Grande Compact Commission* (U.S. Bureau of Reclamation, 2017).

7 Texas Water Development Board (TWDB). *Groundwater Management Areas*, <http://www.twdb.texas.gov/groundwater/management_areas/index.asp>.

8 Comisión Nacional del Agua (CONAGUA). *Suspensión Provisional de Libre Alumbramiento*, <https://sigagis.conagua.gob.mx/gas1/sections/LibreAlumbramiento.html>.

9 U.S. Geological Survey (USGS). *The Transboundary Aquifer Assessment Program (TAAP)*, <https://webapps.usgs.gov/taap/>.

10 Perramond, E. P. The Rise, Fall, and Reconfiguration of the Mexican "Ejido". *Geographical Review.* **98**, 356-371 (2008).

11 IUCN. *Protected Areas Categories*, <https://www.iucn.org/theme/protected-areas/about/protected-area-categories>.

12 U.S. National Park Service. *Big Bend 2009 Fact Sheet* (2009).

13 NACD. *National Association of Conservation Districts Website*, <http://www.nacdnet.org/>.

14 CACD. *Colorado Association of Conservation Districts Website*, <http://www.coloradoacd.org/>.

15 NMACD. *New Mexico Association of Conservation Districts Website*, <http://www.nmacd.org/>.

16 ATSWCD. *Association of Texas Soil and Water Conservation Districts Website*, <http://www.atswcd.org/>.

17 U.S. Department of Agriculture. *Natural Resources Conservation Service Website*, <https://www.nrcs.usda.gov/wps/portal/nrcs/site/national/home/>.

18 COCDA. *Colorado State Conservation Board Webpage*, <https://www.colorado.gov/pacific/agconservation/conservationboard>.

19 NMDA. *New Mexico Department of Agriculture - Agricultural Programs & Resources Webpage*, <http://www.nmda.nmsu.edu/home/divisions/apr/>.

20 TSSWCB. *Texas State Soil and Water Conservation Board Webpage*, <https://www.tsswcb.texas.gov/>.

21 U.S. Fish & Wildlife Service (USFWS). *Landscape Conservation Cooperative network*, <https://lccnetwork.org/>.

22 Migratory Bird Join Ventures Website. *Who we are?* <https://mbjv.org/>.

23 Colorado Water Conservation Board (CWCB). *Instream Flow Program*, <http://cwcb.state.co.us/environment/instream-flow-program/Pages/main.aspx>.

24 Rio Grande Headwaters Land Trust (RiGHT). *Rio Grande Headwaters Land Trust website*, <http://www.riograndelandtrust.org/>.

25 Rio Grande Headwaters Restoration Project. *Rio Grande Headwaters Restoration Project website*, <http://www.riograndeheadwaters.org/>.

26 Middle Rio Grande Endangered Species Collaborative Program (MRGESCP). *Middle Rio Grande Endangered Species Collaborative Program webiste*, <https://webapps.usgs.gov/MRGESCP/>.
