# Supplementary material for: A socio-environmental geodatabase for integrative research in the transboundary Rio Grande/Río Bravo basin
Source: Sci Data. 2020 Mar 6;7:80. doi: 10.1038/s41597-020-0410-1 (PMC7060182; doi:10.1038/s41597-020-0410-1)
Supplement: Supplementary file 2 — Supplementary Information 2 [file 41597_2020_410_MOESM2_ESM.docx]

### Supplementary Information 2: List of Raw Datasets

The five following tables describes the inputs datasets collected to process the output geospatial layers in the five categories: Water & Land Governance (Table 1), Hydrology (Table 2), Water Use & Hydraulic Infrastructures (Table 3), Socio-Economics (Table 4) and Biophysical Environment (Table 5). We provide a direct link to the open-access online source where we downloaded the datasets, except for the datasets shaded in grey for which we addressed a request.

Table 1. List of raw datasets gathered for mapping Water & Land Governance geospatial layers

| **Datasets** | **Output geospatial layers** | **Names of the raw data** | **Description of the raw data** | **Spatial resolution** | **Spatial scope** | **Time Span** | **Date accessed** | **Format** | **Language** | **Source** |
| --- | --- | --- | --- | --- | --- | --- | --- | --- | --- | --- |
| Political Jurisdication Boundaries | Nations | cb_2016_us_nation_5m | U.S. Level 0 Boundary (country) | 1:5,000,000 | US | 2010 | 10/03/2017 | shp | English | U.S. Census Bureau^1^  https://www.census.gov/cgi-bin/geo/shapefiles/index.php |
|  |  | areas_geoestadisticas_estatales | MX Level 0 Boundary (country) | NA | MX | 2016 | 10/4/2017 | shp | Spanish | INEGI^2^  http://www.beta.inegi.org.mx/app/biblioteca/ficha.html?upc=702825217341 |
|  | States | tl_2016_us_state | U.S. Level 1 Boundary (state) | 1:500,000 | US | 2010 | 12/03/2018 | shp | English | U.S. Census Bureau^1^  https://www.census.gov/cgi-bin/geo/shapefiles/index.php |
|  |  | areas_geoestadisticas_estatales | MX Level 1 Boundary (state) | NA | MX | 2016 | 10/4/2017 | shp | Spanish | INEGI^2^  http://www.beta.inegi.org.mx/app/biblioteca/ficha.html?upc=702825217341 |
|  | Counties_and_Municipios | tl_2016_us_county | U.S. Level 2 Boundary (county) | NA | US | 2016 | 13/11/2018 | shp | English | U.S. Census Bureau^1^  https://www.census.gov/cgi-bin/geo/shapefiles/index.php |
|  |  | areas_geoestadisticas_municipales | MX Level 2 Boundary (municipio) | NA | MX | 2016 | 10/04/2017 | shp | Spanish | INEGI^2^  http://www.beta.inegi.org.mx/app/biblioteca/ficha.html?upc=702825217341 |
|  | Places | tl_2016_08_place; tl_2016_35_place; tl_2016_48_place | U.S. Places Boundary (city, town, village) | NA | US | 2016 | 27/07/2017 | shp | English | U.S. Census Bureau^1^  https://www.census.gov/cgi-bin/geo/shapefiles/index.php |
|  |  | poligonos_localidades_urbanas_y_rurales | MX Places Boundary (city, town, village) | NA | MX | 2016 | 10/04/2017 | shp | Spanish | INEGI^2^  http://www.beta.inegi.org.mx/app/biblioteca/ficha.html?upc=702825217341 |
|  | Populated_Places | popPlaces_v2 | Populated Places | NA | North America | 2009 | 31/07/2019 | shp | English | INEGI/NRCan/USGS/CEC^3^  http://www.cec.org/tools-and-resources/north-american-environmental-atlas/map-files |
| Binational Water Management Agencies | Binational_Dams | CO_U; NM_U; TX_U | National Inventory of Dams | NA | US | 2018 | 24/07/2019 | table | English | USACE^4^  http://nid.usace.army.mil/ |
|  | IBWC_Offices | fc_US_OfficeLocations | Location of IBWC offices in the U.S. | NA | US | NA | 29/01/2019 | gdb | English | USIBWC^5^  https://appportal.ibwc.gov/ibwc_geo/public_portal/ |
|  | Canals | fc_US_Canals | Canals |  |  |  |  |  |  |  |
|  | Culverts | fc_US_Culverts | Culverts |  |  |  |  |  |  |  |
|  | DrainageDitches | fc_US_DrainageDitches | Drainage_ditches |  |  |  |  |  |  |  |
|  | Gates | fc_US_Gates | Gates |  |  |  |  |  |  |  |
|  | Laterals | fc_US_Laterals | Laterals |  |  |  |  |  |  |  |
|  | Levees | fc_US_Levees | Levees |  |  |  |  |  |  |  |
|  | MowAreas | fc_US_MowAreas | Mown Areas |  |  |  |  |  |  |  |
|  | Ramps | fc_US_Ramps | Ramp |  |  |  |  |  |  |  |
|  | Ripraps | fc_US_Riprap | Ripraps |  |  |  |  |  |  |  |
|  | ToeDrains | fc_US_ToeDrains | ToeDrains |  |  |  |  |  |  |  |
|  | Wasteways | fc_US_Wasteways | Wasteways |  |  |  |  |  |  |  |
| Federal Water Management Agencies | US_Dams | CO_U; NM_U; TX_U | National Inventory of Dams | NA | US | 2018 | 24/07/2019 | table | English | USACE^4^  http://nid.usace.army.mil/ |
|  | MX_Dams | presas | Location of dams in Mexico | NA | MX | 2015 | 10/04/2017 | shp | Spanish | CENAPRED^6^  http://catalogo.datos.gob.mx/dataset/presas |
| State and Intra-State Water Management Agencies | CDWR_div3 | DIV3CO | Colorado Division of Water Resources boundaries | 1:2,000,000 | CO | 2017 | 31/08/2017 | shp | English | CWCB/DWR^7^  https://www.colorado.gov/pacific/cdss/gis-data-category |
|  | CDWR_div3_districts | Water_Districts | District Boundaries in Colorado | NA | CO | 2017 | 31/08/2017 | shp | English | CWCB/DWR^7^  https://www.colorado.gov/pacific/cdss/gis-data-category |
|  | NMOSE_ WaterMaster_RG | OSE_Administrative_District | District Boundaries for the NM Office of the State Engineer and Interstate Stream Commission | NA | NM | 2017 | 09/06/2017 | shp | English | NMOSE^8^  http://geoSpatialdata-ose.opendata.arcgis.com/datasets/ose-administrative-district |
|  | NM_WaterPlanningRegions_RG | Water_Planning_Regions | Regional Water Planning regions for the NM Office of the State Engineer and Interstate Stream Commission | NA | NM | 2018 | 22/08/2018 | shp | English | NMOSE^8^  http://geoSpatialdata-ose.opendata.arcgis.com/datasets/water-planning-regions |
|  | TCEQ_  service_regions_RG | tceq_regions | 16 TCEQ Service Regions in Texas | NA | TX | 2018 | 26/01/2018 | shp | English | TCEQ^9^  https://www.tceq.texas.gov/gis/download-tceq-gis-data |
|  | TWDB_RWPA_RG | TWDB_RWPAs_2014 | 16 Water Planning Regions in Texas | NA | TX | 2017 | 26/06/2017 | shp | English | TWDB^10^  http://www.twdb.texas.gov/mapping/gisdata.asp |
|  | TWDB_Regional_Project_Team_RG | Regional_Project_Teams | Geographic area assignments for Regional Project Teams | NA | TX | 2018 | 15/11/2018 | shp | English | TWDB^10^  http://www.twdb.texas.gov/mapping/gisdata.asp |
|  | TWDB_IFSS_Offices_RG | IFSS_Offices | Inspection & Field Support Services Offices | NA | TX | 2018 | 15/11/2018 | shp | English | TWDB^10^  http://www.twdb.texas.gov/mapping/gisdata.asp |
|  | MX_State_Conagua_Offices | areas_geoestadisticas_estatales | MX Level 1 Boundary (state) | NA | MX | 2016 | 10/4/2017 | shp | Spanish | INEGI^2^  http://www.beta.inegi.org.mx/app/biblioteca/ficha.html?upc=702825217341 |
|  | JCAS_Chihuahua | areas_geoestadisticas_estatales | MX Level 1 Boundary (state) | NA | MX | 2016 | 10/4/2017 | shp | Spanish | INEGI^2^  http://www.beta.inegi.org.mx/app/biblioteca/ficha.html?upc=702825217341 |
|  | CEAS_Coahuila | areas_geoestadisticas_estatales | MX Level 1 Boundary (state) | NA | MX | 2016 | 10/4/2017 | shp | Spanish | INEGI^2^  http://www.beta.inegi.org.mx/app/biblioteca/ficha.html?upc=702825217341 |
|  | CAED_Durango | areas_geoestadisticas_estatales | MX Level 1 Boundary (state) | NA | MX | 2016 | 10/4/2017 | shp | Spanish | INEGI^2^  http://www.beta.inegi.org.mx/app/biblioteca/ficha.html?upc=702825217341 |
|  | SADM_Nuevo_León | areas_geoestadisticas_estatales | MX Level 1 Boundary (state) | NA | MX | 2016 | 10/4/2017 | shp | Spanish | INEGI^2^  http://www.beta.inegi.org.mx/app/biblioteca/ficha.html?upc=702825217341 |
|  | CEAT_Tamaulipas | areas_geoestadisticas_estatales | MX Level 1 Boundary (state) | NA | MX | 2016 | 10/4/2017 | shp | Spanish | INEGI^2^  http://www.beta.inegi.org.mx/app/biblioteca/ficha.html?upc=702825217341 |
| State and Inter-state Multi-Stakeholder Platforms | ***Rio Grande Compact*** | | | | | | | | | |
|  | Interstate_Compacts | WBDHU10 | National Watershed Boundary Dataset | 1:100,000 | US | 2013 | 05/03/2017 | shp | English | USGS & USDA^11^  <https://www.usgs.gov/core-science-systems/ngp/national-hydrography/access-national-hydrography-products> |
|  | RG_Compact_Gauges | USGS_Streamgages-NHD_Locations | Location of Stream Flow Gages operated by USGS | 1:100,000 | US | 2002 | 16/11/2018 | mdb | English | USGS^12^  https://water.usgs.gov/GIS/metadata/usgswrd/XML/streamgages.xml#stdorder |
|  | RG_Compact_Dams | CO_U; NM_U; TX_U | National Inventory of Dams | NA | US | 2018 | 24/07/2019 | table | English | USACE^4^  http://nid.usace.army.mil/ |
|  | ***San Juan Chama Project-SJCP*** | | | | | | | | | |
|  | SJCP_Tunnel | NHDFlowline, NHDFLowline | Flow network consisting predominantly of stream/river and artificial path vector features | 1:100,000 | US | 2002 | 05/03/2017 | shp | English | USGS-NHD^13^  https://www.usgs.gov/core-science-systems/ngp/national-hydrography |
|  | SJCP_Reservoirs | NHDWaterbody | Areal NHD hydrographic waterbody features, such as LakePond, Reservoir, and Estuary | 1:100,000 | US | 2002 | 05/03/2017 | shp | English | USGS-NHD^13^  https://www.usgs.gov/core-science-systems/ngp/national-hydrography |
|  | SJCP_Users | NM_irrigation_Districts | Irrigation Districts in new Mexico | NA | NM | 2017 | 09/06/2017 | shp | Spanish | NMOSE^8^  http://geoSpatialdata-ose.opendata.arcgis.com/datasets/nm-irrigation-districts |
|  |  | tl_2016_us_county | U.S. Level 2 Boundary (county) | NA | US | 2016 | 13/11/2018 | shp | English | U.S. Census Bureau^1^  https://www.census.gov/cgi-bin/geo/shapefiles/index.php |
|  |  | tl_2016_35_place | Places in New Mexico | NA | NM | 2016 | 27/07/2017 | shp | English | U.S. Census Bureau^1^  https://www.census.gov/cgi-bin/geo/shapefiles/index.php |
|  |  | tl_2016_us_aiannh | American Indian Area Geography | NA | US | 2016 | 27/07/2017 | shp | English | U.S. Census Bureau^1^  https://www.census.gov/cgi-bin/geo/shapefiles/index.php |
|  |  | NHDWaterbody | Areal NHD hydrographic waterbody features, such as LakePond, Reservoir, and Estuary | 1:100,000 | CO, NM | 2002 | 05/03/2017 | shp | English | USGS-NHD^13^  https://www.usgs.gov/core-science-systems/ngp/national-hydrography |
|  | ***Rio Grande Project*** | | | | | | | | | |
|  | RG_Project_Dams | CO_U; NM_U; TX_U | National Inventory of Dams | NA | US | 2018 | 24/07/2019 | table | English | USACE^4^  http://nid.usace.army.mil/ |
|  | RG_Project_IrrigDistricts | NM_Irrigation_Districts | NM Irrigation Districts | NA | NM | 2017 | 09/06/2017 | shp | English | NMOSE^8^  http://geoSpatialdata-ose.opendata.arcgis.com/datasets/nm-irrigation-districts |
|  |  | TCEQ_WATER_DISTRICTS | Location of water districts within the state of Texas | NA | TX | 2015 | 26/01/2018 | shp | English | TCEQ^9^  https://www.tceq.texas.gov/gis/download-tceq-gis-data |
|  |  | Distritos_riego_2015_2016  Distritos_riego_2016_2017 | Location of irrigation districts in Mexico and water use in 2015-2016 and 2016-2017 | 1:250,000 | MX | 2015-2016, 2016-2017 | 23/02/2018; 03/09/2018 | shp | Spanish | CONAGUA^14^  http://sina.conagua.gob.mx/sina/tema.php?tema=distritosriego |
|  | ***Consejo de Cuenca del Río Bravo*** | | | | | | | | | |
|  | MX_Consejo_Cuenca | rha250kgw | Hydrological-Administrative region (VI) of the Río Bravo | 1:250,000 | MX | 2007 | 06/09/2017 | shp | Spanish | CONAGUA^15^  http://www.conabio.gob.mx/informacion/gis/ |
|  | ***Rio Grande Roundtable*** | | | | | | | | | |
|  | CO_RG_Roundtable | tl_2016_us_state | U.S. Level 1 Boundary (state) | 1:500,000 | US | 2010 | 12/03/2018 | shp | English | U.S. Census Bureau^1^  https://www.census.gov/cgi-bin/geo/shapefiles/index.php |
| Groundwater Focused Institutions | ***Colorado*** | | | | | | | | | |
|  | CO_RGWCD_bound | District_Boundary | Boundary of Rio Grande Water Conservation Districts (RGWCD) | NA | CO | 2018 | 27/11/2018 | shp | English | RGWCD  Shared dataset - No download link |
|  | CO_ClosedBasin_bound | ClosedBasinBoundary | Boundary of the Closed Basin | NA | CO | NA | 08/06/2017 | shp | English | CWCB/DWR^7^  https://www.colorado.gov/pacific/cdss/gis-data-category |
|  | CO_RGWCD_Wells | RGWCD_confined_well  RGWCD_unconfined_well | Confined and unconfined wells monitored by RGWCD | NA | CO | 2017 | 31/08/2017 | kmz | English | RGWCD^16^  http://www.rgwcd.org/well-information |
|  | CO_Subdistricts_bound | Subdistrict_1_bndry2006Mar_polygon  SD2_Boundary  SD3_Boundary  SD4_Boundary  SD5_Boundary  SD6_Boundary | Boundaries of Subdistrict #1, #2, #3, #4, #5, #6 | NA | CO | 2006  -  2018 | 27/11/2018 | shp | English | RGWCD  Shared dataset - No download link |
|  | CO_Augment_replacement_plans | DWR_Water_Right_-_Net_Amounts | Surface and Ground Water Rights in Colorado | NA | CO | 2019 | 02/08/2019 | table | English | CDWR^17^  [https://data.colorado.gov/Water/DWR-Water-Right-Net-Amounts/acsg-f33s/data](https://data.colorado.gov/Water/DWR-Water-Right-Net-Amounts/acsg-f33s/data%20) |
|  | ***New Mexico*** | | | | | | | | | |
|  | NM_declared_GW_basins | Declared_Groundwater_Basins | Declared Groundwater Basins in New Mexico | NA | NM | 2017 | 09/06/2017 | shp | English | NMOSE^8^  http://geoSpatialdata-ose.opendata.arcgis.com/datasets/ose-declared-groundwater-basins |
|  | ***Texas*** | | | | | | | | | |
|  | TX_GMA | Groundwater_Management_Areas_082615 | Groundwater Management Areas in Texas | NA | TX | 2015 | 26/06/2017 | shp | English | TWDB^10^  http://www.twdb.texas.gov/mapping/gisdata.asp |
|  | TX_GCD | TCEQ_GCD | Groundwater Conservation Districts in Texas | NA | TX | 2015 | 28/07/2017 | shp | English | TCEQ^9^  https://www.tceq.texas.gov/gis/download-tceq-gis-data |
|  | TX_PMGA | TCEQ_PGMA | Priority Groundwater Management Areas in Texas | NA | TX | 2017 | 28/07/2017 | shp | English | TCEQ^9^  https://www.tceq.texas.gov/gis/download-tceq-gis-data |
|  | ***Transboundary*** | | | | | | | | | |
|  | TAAP_Aquifers | Mesilla_ConejosMedanos_pg.shp | Boundary of the Mesilla/Conejos-Médanos aquifer | NA | US - MX | NA | 06/01/2020 | shp | English | Teeple (2017)^18^  [https://doi.org/10.3133/sir20175028](https://urldefense.proofpoint.com/v2/url?u=https-3A__doi.org_10.3133_sir20175028&d=DwMF-g&c=qKdtBuuu6dQK9MsRUVJ2DPXW6oayO8fu4TfEHS8sGNk&r=zCIn5D2H25roPkfApUnV62NaVogkWhHAPX55BiXrmqk&m=b-fXSEWWArR071fS9LeIqQhid_6NjLaeC9zeCZtEcQ0&s=7-kdZueO0ypYquw7yGreM05G8gd2m5ikawohbXD1ICI&e=) |
|  |  | Hueco.shp | Boundary of the Hueco Bolson aquifer | NA | US - MX | NA | 07/01/2020 | shp | English | Driscoll & Sherson (2016)^19^  <http://dx.doi.org/10.3133/sir20165006> |
| Irrigation Management Agencies | CO_Irrigation_Organizations | Div3_Ditch_Service_Post99 | Colorado irrigation districts, ditch companies and acequias boundaries | NA | CO | 2015 | 08/06/2017 | shp | English | CWCB/DWR^7^  https://www.colorado.gov/pacific/cdss/division-3-rio-grande |
|  | NM_Irrigation_Districts | NM_irrigation_Districts | New Mexico irrigation districts’ boundaries | NA | NM | 2017 | 09/06/2017 | shp | English | NMOSE^8^  http://geoSpatialdata-ose.opendata.arcgis.com/datasets/nm-irrigation-districts |
|  | TX_Water_Districts | TCEQ_WATER_DISTRICTS | TCEQ water districts’ boundaries | NA | TX | 2015 | 26/01/2018 | shp | English | TCEQ^9^  https://www.tceq.texas.gov/gis/download-tceq-gis-data |
|  | MX_Distritos_Riego | Distritos_riego_2015_2016  Distritos_riego_2016_2017 | Location of irrigation districts in Mexico and water use in 2015-2016 and 2016-2017 | 1:250,000 | MX | 2015-2016, 2016-2017 | 23/02/2018; 03/09/2018 | shp | Spanish | CONAGUA^14^  http://sina.conagua.gob.mx/sina/tema.php?tema=distritosriego |
| Land Management | Land_Management | BLM_National_Surface_Management_Agency.lpk | Public and Private domains outlines in U.S. | 1:24,000 to 1:100,000 | US | 2017 | 08/06/2017 | ArcGIS Layer Package | English | BLM^20^  https://landscape.blm.gov/geoportal/catalog/search/resource/details.page?uuid=%7B2A8B8906-7711-4AF7-9510-C6C7FD991177%7D |
|  |  | PERIMETRAL_CHIH  PERIMETRAL_COAH  PERIMETRAL_DGO  PERIMETRAL_NL  PERIMETRAL_TAMPS | Certified Ejidos and Communal Lands Outlines in Coahuila, Chihuahua, Durnago, Nueva leon, Tamaualipas | NA | MX | 2019 | 31/07/2019 | shp | Spanish | RAN^21^  <http://datos.ran.gob.mx/conjuntoDatosPublico.php> |
| Protected Areas | ProtectedAreas | CEC_NA_PAD_2017 | North American Terrestrial and Marine Protected Areas, 2017 | NA | North America | 2017 | 31/07/2019 | shp | English | CEC/CONANP/CARTS/ MDDELCC/ USGS^22^  http://www.cec.org/tools-and-resources/north-american-environmental-atlas/map-files |
| Border Control | Border_Crossings | Canada_and_Mexico_Border_Crossings. | Canada and Mexico Border Crossings | 1:1,000,000 | US-MX | 2011 | 08/05/2018 | shp | English | HIFLD^23^  https://hifld-geoplatform.opendata.arcgis.com/datasets/canada-and-mexico-border-crossings |
|  | Border_Fences | Border_fence_map | Border Fences | NA | US-MX | 2016 | 27/07/2017 | shp | English | The Center for Investigative Reporting  https://github.com/cirlabs/border_fence_map |
|  | Border_Sectors | SwBorderSector | Southwest Border Patrol Sector Map | NA | US | 2018 | 09/05/2018 | shp | English | HSIP^24^  <https://www.arcgis.com/home/item.html?id=e4c86699f8b84b6f9e5b26bf452323a8> |
|  |  |  | Southwest Border Migration Apprehension Statistics | NA | US | 2017-2018 | 26/07/2019 | table | English | U.S. Customs and Border Protection^25^  <https://www.cbp.gov/newsroom/stats/usbp-sw-border-apprehensions> |
| Soil and Water Conservation Districts | SWCD | Conservation_Individual_Districts | Colorado Soil and Water Conservation Districts boundaries | NA | CO | NA | 18/01/2018 | shp | English | CSCB  Shared dataset - No download link |
|  |  | NM_Soil_Water_Conservation_Districts | New Mexico Soil and Water Conservation Districts boundaries | NA | NM | 2017 | 24/01/2019 | shp | English | NMOSE^8^  http://geoSpatialdata-ose.opendata.arcgis.com/datasets/nm-soil-water-conservation-districts |
|  |  | Rio_Grande_SWCD | Texas Soil and Water Conservation Districts boundaries | NA | TX | 2018 | 19/01/2018 | shp | English | TSSWCB  Shared dataset - No download link |
| Conservation projects | ***Transboundary*** | | | | | | | | | |
|  | LCC_network | FWS_LCC | Landscape Conservation Cooperative Boundaries | NA | North America | 2015 | 15/02/2019 | shp | English | USFWS^26^  https://www.sciencebase.gov/catalog/item/55b943ade4b09a3b01b65d78 |
|  | Bird_Conservation_JoinVentures | North_American_Joint_Ventures_Web_Mercator_080317_Revision | North American Bird Conservation Joint Ventures | NA | US | 2017 | 14/02/2019 | shp | English | USFWS^27^  https://ecos.fws.gov/ServCat/Reference/Profile/81433 |
|  | ***Colorado – Instream Flow Program*** | | | | | | | | | |
|  | CO_ISF_Reaches | ISF_Reaches_Decreed  ISF_Reaches_Pending | Instream Flow Reaches - Decreed and pending reaches | NA | CO | 2017 | 13/03/2018 | shp | English | CWCB/DWR^7^  https://www.colorado.gov/pacific/cdss/gis-data-category |
|  | CO_ISF_Termini | ISF_Termini_Decreed  ISF_Termini_Pending | Instream Flow Termini - Decreed and pending termini points | NA | CO | 2017 | 13/03/2018 | shp | English | CWCB/DWR^7^  https://www.colorado.gov/pacific/cdss/gis-data-category |
|  | CO_Lakes | Lakes | Natural Lake Levels - Decreed Lakes | NA | CO | 2015 | 13/03/2018 | shp | English | CWCB/DWR^7^  https://www.colorado.gov/pacific/cdss/gis-data-category |

Table 2. List of raw datasets gathered for mapping “Hydrology” geospatial layers

| **Datasets** | **Output geospatial layers** | **Names of the raw data** | **Description of the raw data** | **Spatial resolution** | **Spatial scope** | **Time Span** | **Date accessed** | **Format** | **Language** | **Source** |
| --- | --- | --- | --- | --- | --- | --- | --- | --- | --- | --- |
| Watershed boundary | Sub_Basins  RGB_Basin | hydrobasins_centralam | Watershed boundaries derived from HydroSHEDS | 15 arc-second | Central America | NA | 09/06/2016 | shp | English | FAO^28^  <https://geonetwork-opensource.org/docs.html> |
| River networks | Rivers | rivers_centam_37249 | River network derived from HydroSHEDS | 15 arc-second | Central America | NA | 01/08/2019 | shp | English | FAO^29^  <https://geonetwork-opensource.org/docs.html> |
| Aquifer boundaries | Aquifer | aquifrp025 | Shallowest principal aquifers boundaries in U.S. | 1:2,500,000 | US | NA | 20/12/2017 | shp | English | USGS^30^  https://catalog.data.gov/dataset/aquifers |
|  |  | Acuiferos_disponibilidad_2015 | Aquifer Boundaries in Mexico | NA | MX | NA | 07/03/2018 | shp | Spanish | CONAGUA^14^  http://sina.conagua.gob.mx/sina/tema.php?tema=acuiferos |
| Gauging stations | Gauges_usgs | USGS_Streamgages-NHD_Locations | Location of Stream Flow Gages monitored by USGS in U.S. | 1:100,000 | US | 2006 | 16/11/2018 | shp | English | USGS^12^  https://water.usgs.gov/GIS/metadata/usgswrd/XML/streamgages.xml#stdorder |
|  | Gauges_ibwc | fc_US_WQMonStations_SFGages | Location of Stream Flow Gages monitored by IBWC along the U.S.-MX border | NA | Border | 2010 | 26/05/2017 | gdb | English | USIBWC^5^  https://appportal.ibwc.gov/ibwc_geo/public_portal/ |
|  | Gauges_conagua | estacionesMX | Location of Stream Flow Gages monitored by CONAGUA in Mexico | NA | MX | NA | 06/12/2017 | table | Spanish | CONAGUA^31^  ftp://ftp.conagua.gob.mx/Bandas/Bases_Datos_Bandas |
| Quality Stations | Quality_Stations | fc_US_WQMonStations_SFGages | Location of Water Quality Monitoring Stations and Stream Flow Gages monitored by IBWC and TCEQ | NA | US | 2008 | 29/01/2019 | gdb | English | USIBWC^5^  https://appportal.ibwc.gov/ibwc_geo/public_portal/ |

Table 3. List of raw datasets gathered for mapping “Water Use & Hydraulic Infrastructures” geospatial layers

| **Datasets** | **Output geospatial layers** | **Names of the raw data** | **Description of the raw data** | **Spatial resolution** | **Spatial scope** | **Time Span** | **Date accessed** | **Format** | **Language** | **Source** |
| --- | --- | --- | --- | --- | --- | --- | --- | --- | --- | --- |
| Withdrawals | Withdrawals_2015 | usco2015v2.0 | Estimated Use of Water in the United States County-Level Data | county | US | 2015 | 31/07/2019 | table | English | USGS^32^  <https://water.usgs.gov/watuse/data/data2015.html> |
|  |  | Fuente_usos_consuntivos_municipio_2015  Intensidad_usos_consuntivos_municipio_2015 | Water Use in Mexico Municipio-Level Data | municipio | MX | 2015 | 04/08/2019 | shp | Spanish | CONAGUA^14^  <http://sina.conagua.gob.mx/sina/tema.php?tema=usosAgua> |
| Irrigation | Irrigation_US  _US | usco2015v2.0 | Spatial coverage of irrigated lands and irrigation systems (surface, sprinkler micro-irrigation) in acres | county | US | 2015 | 31/07/2019 | table | English | USGS^32^  <https://water.usgs.gov/watuse/data/data2015.html> |
|  | Irrigation_MX | cag_2007_15, cag_2007_16, cag_2007_17 | Spatial coverage of irrigated lands in hectares, irrigation systems in number of farms (lined, earthen canal, sprinkler, micro-sprinkler, drip, other), and source of water in number of farms (reservoir, rainwater catchment, wells, springs, dams) | municipio | MX | 2007 | 23/06/2017 | table | Spanish | INEGI^33^  <https://www.inegi.org.mx/temas/agricultura/default.html#Tabulados> |
| Water Rights | CO_WaterRights | DWR_Water_Right_-_Net_Amounts | Surface and Ground Water Rights in Colorado | NA | CO | 2019 | 02/08/2019 | table | English | CDWR^17^  [https://data.colorado.gov/Water/DWR-Water-Right-Net-Amounts/acsg-f33s/data](https://data.colorado.gov/Water/DWR-Water-Right-Net-Amounts/acsg-f33s/data%20) |
|  | NM_WaterRights | OSE_Points_of_Diversion | Points of diversion in New Mexico | NA | NM | 2018 | 08/11/2018 | shp | English | NMOSE^8^  http://geoSpatialdata-ose.opendata.arcgis.com/datasets/ose-points-of-diversion |
|  | TX_WaterRights | Rio_Grande_unverified_WaterRights | Surface Water Rights in Texas | NA | TX | 2017 | 20/06/2017 | shp | English | TCEQ^34^  https://www.tceq.texas.gov/permitting/water_rights/wr_technical-resources/wam.html/#wrapinput |
|  | CHI_WaterRights | Chihuahua  Aprovechamientos_Superficiales | Surface Water Rights in Mexico and Ground Water Rights in Chihuahua | NA | MX | NA | 10/04/2017 | kmz | Spanish | CONAGUA^35^  http://siga.conagua.gob.mx/REPDA/Menu/MenuKMZ.html |
|  | COA_WaterRights | Coahuila  Aprovechamientos_Superficiales | Surface Water Rights in Mexico and Ground Water Rights in Coahuila | NA | MX | NA | 10/04/2017 | kmz | Spanish | CONAGUA^35^  http://siga.conagua.gob.mx/REPDA/Menu/MenuKMZ.html |
|  | DUR_WaterRights | Durango  Aprovechamientos_Superficiales | Surface Water Rights in Mexico and Ground Water Rights in Durango | NA | MX | NA | 10/04/2017 | kmz | Spanish | CONAGUA^35^  http://siga.conagua.gob.mx/REPDA/Menu/MenuKMZ.html |
|  | NVL_WaterRights | Nuevo_Leon  Aprovechamientos_Superficiales | Surface Water Rights in Mexico and Ground Water Rights in Nuevo León | NA | MX | NA | 10/04/2017 | kmz | Spanish | CONAGUA^35^  http://siga.conagua.gob.mx/REPDA/Menu/MenuKMZ.html |
|  | TAM_WaterRights | Tamaulipas  Aprovechamientos_Superficiales | Surface Water Rights in Mexico and Ground Water Rights in Tamaulipas | NA | MX | NA | 10/04/2017 | kmz | Spanish | CONAGUA^35^  http://siga.conagua.gob.mx/REPDA/Menu/MenuKMZ.html |
| Dams and reservoirs | Dams_us | CO_U; NM_U; TX_U | National Inventory of Dams | NA | US | 2018 | 24/07/2019 | table | English | USACE^4^  http://nid.usace.army.mil/ |
|  | Dams_mx | presas | Dams in Mexico | NA | MX | NA | 10/04/2017 | shp | Spanish | CENAPRED^6^  http://catalogo.datos.gob.mx/dataset/presas |
| Water diversion structures | Diversion_US | NHDFlowline | Linear surface water drainage network (stream network, artificial path, canal/ditch, pipeline, connector, underground conduit, and coastline) in U.S., derived from USNHD | 1:100,000 | US | 2002 | 05/03/2017 | shp | English | USGS-NHD^13^  https://www.usgs.gov/core-science-systems/ngp/national-hydrography |
|  | Diversion_MX | Acueducto,  Canals | Canals and aqueducts in Mexico | 1:1,000,000 | MX | 2000 | 10/04/2017 | shp | Spanish | INEGI^36^  https://www.inegi.org.mx/app/biblioteca/ficha.html?upc=702825267643 |
|  | Diversion_CO | Div3_Canals | Division 3 Canals - Irrigation canals and ditches | NA | CO | 2015 | 31/08/2017 | shp | English | CWCB/DWR^7^  https://www.colorado.gov/pacific/cdss/division-3-rio-grande |
| Wells | Wells_us_nhd | NHDPoint | Wells location derived from USNHD | 1:100,000 | US | 2017 | 05/03/2017 | shp | English | USGS-NHD^13^  https://www.usgs.gov/core-science-systems/ngp/national-hydrography |
|  | Wells_co_cwcb | WellPermitPublic | Dataset of well permits in Colorado from CDWR database | NA | CO | 2019 | 06/08/2019 | shp | English | CWCB/DWR^7^  https://www.colorado.gov/pacific/cdss/gis-data-category |
|  | Wells_nm_nmose | OSE_Points_of_Diversion | Points of diversion in New Mexico from NMOSE database | NA | NM | 2018 | 08/11/2018 | shp | English | NMOSE^8^  http://geoSpatialdata-ose.opendata.arcgis.com/datasets/ose-points-of-diversion |
|  | Wells_tx_twdb | TWDB_Groundwater | Well Locations in Texas from TWDB Groundwater Database | NA | TX | 2019 | 06/08/2019 | shp | English | TWDB^10^  http://www.twdb.texas.gov/mapping/gisdata.asp |

Table 4. List of raw datasets gathered for mapping “Socio-Economic” geospatial layers

| **Datasets** | **Output geospatial layers** | **Names of the raw data** | **Description of the raw data** | **Spatial resolution** | **Spatial scope** | **Time Span** | **Date accessed** | **Format** | **Language** | **Source** |
| --- | --- | --- | --- | --- | --- | --- | --- | --- | --- | --- |
| Population | Population | counties | 1990 Census Gazetteer Files at the county level | county | US | 1990 | 27/04/2018 | ASCII text file | English | US Census Bureau^37^  https://www.census.gov/geo/maps-data/data/gazetteer1990.html |
|  |  | DEC_00_SF1_DP1_with_ann | Profile of General Demographic Characteristics: 2000 at the county level | county | US | 2000 | 26/04/2018 | table | English | US Census Bureau^37^  <https://factfinder.census.gov/faces/nav/jsf/pages/index.xhtml> |
|  |  | DEC_10_DP_DPDP1_with_ann | Profile of General Population and Housing Characteristics: 2010. 2010 Demographic Profile Data at the county level | county | US | 2010 | 26/04/2018 | table | English | US Census Bureau^37^  <https://factfinder.census.gov/faces/nav/jsf/pages/index.xhtml> |
|  |  | CPyV90_Chih_Poblacion  CPyV90_Coah_Poblacion  CPyV90_Dgo_Poblacion  CPyV90_NL_Poblacion  CPyV90_Tamps_Poblacion | Historical census on population at the municipio level, MX, 1990 | municipio | MX | 1990 | 04/12/2019 | shp | Spanish | INEGI^38^  <https://www.inegi.org.mx/programas/ccpv/cpvsh/> |
|  |  | CPyV2000_Chih_Poblacion  CPyV2000_Coah_Poblacion  CPyV2000_Dgo_Poblacion  CPyV2000_NL_Poblacion  CPyV2000_Tamps_Poblacion | Historical census on population at the municipio level, MX, 2000 | municipio | MX | 2000 | 04/12/2019 | shp | Spanish | INEGI^38^  <https://www.inegi.org.mx/programas/ccpv/cpvsh/> |
|  |  | 01_01B_MUNICIPAL_05  01_01B_MUNICIPAL_08  01_01B_MUNICIPAL_10  01_01B_MUNICIPAL_19  01_01B_MUNICIPAL_28 | Historical census on population at the municipio level, MX, 2010 | municipio | MX | 2010 | 04/12/2019 | shp | Spanish | INEGI^38^  <https://www.inegi.org.mx/programas/ccpv/cpvsh/> |
| Population density | popdens_00  popdens_05  popdens_10  popdens_15  popdens_20 | gpw_v4_population_density_rev11_yyyy_30_sec  yyyy = [2000, 2005, 2010, 2015, 2020] | Gridded population of the world, Population Density, v4.11 (2000, 2005, 2010, 2015, 2020) | 30 arc second | Global | 2000, 2005, 2010, 2015, 2020 | 31/07/2019 | raster | English | CIESIN - Columbia University^39^  <https://sedac.ciesin.columbia.edu/data/set/gpw-v4-population-density-rev11> |
| Income | Income_US | CA1_1969_2015__ALL_AREAS | Personal income at the county level, U.S. | county | US | 1969-2015 | 10/09/2017 | table | English | USBEA^40^  https://www.bea.gov/regional/downloadzip.cfm |
|  | DistributionIncome_MX90 | ingmun90gw | Income distribution at the municipio level, MX, 1990 | municipio | MX | 1990 | 10/04/2017 | shp | Spanish | INEGI compiled by CONABIO^41^  http://www.conabio.gob.mx/informacion/gis/ |
|  | DistributionIncome_MX00 | ingmun00gw | Income distribution at the municipio level, MX, 2000 | municipio | MX | 2000 | 10/04/2017 | shp | Spanish | INEGI compiled by CONABIO^41^  http://www.conabio.gob.mx/informacion/gis/ |
|  | DistributionIncome_MX10 | ingmun10gw | Income distribution at the municipio level, MX, 2010 | municipio | MX | 2010 | 10/04/2017 | shp | Spanish | INEGI compiled by CONABIO^41^  http://www.conabio.gob.mx/informacion/gis/ |
| Number and size of farms | Farms_US2007  Farms_US2012 | Colorado: cov1.txt;  New Mexico: nmv1.txt;  Texas: txv1a.txt | Agricultural Census full report | county | US | 2007, 2012 | 14/02/2018 | txt | English | USDA-NASS^42^  https://www.nass.usda.gov/AgCensus/index.php |
|  | Farms_MX2007 | cag_2007_01 | Farms number and area at the municipio level, MX, 2007 | municipio | MX | 2007 | 23/06/2017 | table | Spanish | INEGI^33^  <https://datos.gob.mx/busca/dataset/censo-agricola-ganadero-y-forestal-2007> |
| Transport infrastructures | Roads | gROADS-v1-americas | Global Roads Open Access Data Set, Version 1 (gROADSv1) | NA | Global | 1980 to 2010 | 10/08/2017 | shp | English | CIESIN - Columbia University/ ITOS - University of Georgia^43^  http://sedac.ciesin.columbia.edu/data/set/groads-global-roads-open-access-v1 |
|  | Railroads | railroads_l_v2 | Railroads of North America | NA | North America | 2009 | 31/07/2019 | shp | English | INEGI/NRCan/USGS/CEC^44^  http://www.cec.org/tools-and-resources/north-american-environmental-atlas/map-files |

Table 5. List of raw datasets gathered for mapping “Biophysical Environment” geospatial layers

| **Datasets** | **Output geospatial layers** | **Names of the raw data** | **Description of the raw data** | **Spatial resolution** | **Spatial scope** | **Time Span** | **Date accessed** | **Format** | **Language** | **Source** |
| --- | --- | --- | --- | --- | --- | --- | --- | --- | --- | --- |
| Ecoregions | NA_ECO_3 | NA_CEC_Eco_Level3 | Level III ecoregions of North America | 1:250,000 | North America | 2010 | 25/01/2019 | shp | English | US-EPA^45^  https://www.epa.gov/eco-research/level-iii-and-iv-ecoregions-continental-united-states |
|  | US_ECO_4 | us_eco_l4_no_st | Level IV ecoregions of the Conterminous United States | 1:250,000 | US | 2013 | 25/01/2019 | shp | English | US-EPA^46^  https://www.epa.gov/eco-research/level-iii-and-iv-ecoregions-continental-united-states |
| Habitat | ***US*** | | | | | | | | | |
|  | US_Crithab_Line,  US_Crithab_Poly | CRITHAB_POLY, CRITHAB_LINE | U.S. FWS Threatened & Endangered Species Active Critical Habitat Boundaries | NA | US | 2019 | 14/02/2019 | shp | English | USFWS^47^  https://ecos.fws.gov/ecp/report/table/critical-habitat.html |
|  | US_Wetlands US_Riparian | CO_shapefile_wetlands, NM_shapefile_wetlands, TX_shapefile_wetlands | National Wetland Inventory (wetlands and riparian areas) | 1:24,000 | US | 2018 | 14/02/2019 | shp | English | USFWS^48,49^  https://www.fws.gov/wetlands/Data/Data-Download.html |
|  | ***Mexico*** | | | | | | | | | |
|  | MX_Ramsar_Site | Sitios_Ramsar_Geo_ITRF92_2015 | Ramsar sites in Mexico | NA | MX | 2015 | 05/12/2019 | shp | Spanish | CONANP^50^  https://datos.gob.mx/busca/dataset/coberturas-para-manejadores-de-sig |
|  | MX_SAP | sap_gw | Sitios de atención prioritaria para la conservación de la biodiversidad | 1:1,000,000 | MX | 2016 | 05/12/2019 | shp | Spanish | CONABIO^51^  <http://www.conabio.gob.mx/informacion/gis/> |
|  | MX_SPR | spr_gw | Sitios prioritarios para la restauración | 1:1,000,000 | MX | 2016 | 05/12/2019 | shp | Spanish | CONABIO^52^  <http://www.conabio.gob.mx/informacion/gis/> |
| Soil Cover | soil | dsmw | Digital Soil Map of the World (FAO taxonomy) | 1:5,000,000 | Global | 2007 | 22/09/2016 | shp | English | FAO^53,54^  http://www.fao.org/geonetwork/srv/en/metadata.show?id=14116 |
| Elevation | elev | GloElev_30as.rar | Harmonized World Soil Database (version 1.1)  Global elevation derived from the SRTM 30-second DEM and the USGS GTOPO30 | 30 arc second | Global | NA | 11/05/2017 | raster | English | FAO/IIASA/ISRIC/ISS-CAS/JRC^55^  http://www.fao.org/soils-portal/soil-survey/soil-maps-and-databases/harmonized-world-soil-database-v12/en/ |
| Slope | slopedeg |  |  |  |  |  |  |  |  |  |
|  | slopepct |  |  |  |  |  |  |  |  |  |
| Land Cover | lc2010 | NA_NALCMS_LC_30m_LAEA_mmu12_urb05 | North American land cover in 2010 | 30 m | North America | 2010 | 31/07/2019 | raster | English | CCRS/CCMEO/NRCan/CONABIO/CONAFOR/INEGI/USGS^56^  http://www.cec.org/tools-and-resources/north-american-environmental-atlas/map-files |
| Land Use | cdl2008  cdl2009  cdl2010  cdl2011  cdl2012  cdl2013  cdl2014  cdl2015  cdl2016  cdl2017  cdl2018 | CDL_yyyy_08, CDL_yyyy _35, CDL_yyyy_48,  yyyy = [2008, 2009, 2010, 2011, 2012, 2013, 2014, 2015, 2016, 2017, 2018] | Time-series (2008-2018) of the Cropland Data Layer in US | 30 m | U.S. | 2008 – 2018 | 16/02/2019 | raster | English | USDA-NASS^57^  https://nassgeodata.gmu.edu/CropScape/ |
|  | MX_LU_2011  MX_LU_2014 | usv250s5_union  usv250s6_union | Land-Use in Mexico in 2011 and 2014 | 30 m | Mexico | 2011, 2014 | 2011:  10/04/2017  2014:  22/08/2018 | shp | Spanish | INEGI^58,59^  https://www.inegi.org.mx/temas/usosuelo/ |
|  | MX_ancrop_winter07  MX_ancrop_spring07  MX_percrop07 | cag_2007_08, cag_2007_08a, cag_2007_09, cag_2007_10, cag_2007_10a | Annual Winter, Annual Spring and Perennial Areas Planted in Mexico in 2007 | county | Mexico | 2007 | 16/05/2017 | table | Spanish | INEGI^33^  https://www.inegi.org.mx/programas/cagf/2007/default.html |

### References

1 U.S. Census Bureau. 2016 TIGER/Line® Shapefiles, <https://www.census.gov/> (2016).

2 Instituto Nacional de Estadística y Geografía (INEGI). Marco Geoestadístico, [http://www.beta.inegi.org.mx/app/biblioteca/ficha.html?upc=702825217341](http://www.beta.inegi.org.mx/app/biblioteca/ficha.html?upc=702825217341%20) (2016).

3 Instituto Nacional de Estadística y Geografía (INEGI), Natural Resources Canada (NRCan), U.S. Geological Survey (USGS) & Commission for Environmental Cooperation (CEC). North American Environmental Atlas - Populated Places, 2009, <http://www.cec.org/tools-and-resources/north-american-environmental-atlas/map-files> (2010).

4 U.S. Army of Corps Engineer (USACE). 2018 National Inventory of Dams (NID), <http://nid.usace.army.mil/> (2019).

5 U.S. International Boundary and Water Commissions (IBWC). *GIS Portal,* <https://appportal.ibwc.gov/ibwc_geo/public_portal/> (2018).

6 Centro Nacional de Prevención de Desastres (CENAPRED). Presas, <http://catalogo.datos.gob.mx/dataset/presas> (2015).

7 Colorado Water Conservation Board (CWCB) & Division of Water Resources (DWR). *Colorados' Decision Support Systems*, <https://www.colorado.gov/pacific/cdss/gis-data-category> (2014).

8 New Mexico Office of the State Engineer (NMOSE). *Open Data Site*, <http://geospatialdata-ose.opendata.arcgis.com/> (2018).

9 Texas Commission on Environmental Quality (TCEQ). *GIS Data*, <https://www.tceq.texas.gov/gis/download-tceq-gis-data> (2018).

10 Texas Water Development Board (TWDB). *GIS Datasets*, <http://www.twdb.texas.gov/mapping/gisdata.asp> (2018).

11 U.S. Geological Survey & U.S. Department of Agriculture Natural Resources Conservation Service. *Federal Standards and Procedures for the National Watershed Boundary Dataset (WBD) (4 ed.)*. Techniques and Methods 11–A3 (U.S. Geological Survey, 2013).

12 Stewart, D. W., Rea, A. & Wolock, D. M. USGS Streamgages Linked to the Medium Resolution NHD, <https://doi.org/10.3133/ds195> (2006).

13 U.S. Geological Survey (USGS). National Hydrography Dataset – Medium resolution (1:100,000), <https://www.usgs.gov/core-science-systems/ngp/national-hydrography> (2014).

14 Comisión Nacional del Agua (CONAGUA). Sistema Nacional de Información del Agua (SINA), <http://201.116.60.25/sina/> (2018).

15 Comisión Nacional del Agua (CONAGUA). Regiones Hidrológicas Administrativas. (Organismos de Cuenca). Shapefile. Escala: 1:250000, <http://www.conabio.gob.mx/informacion/gis/> (2007).

16 Rio Grande Water Conservation District (RGWCD). Well information, <https://www.rgwcd.org/well-information>.

17 Colorado Division of Water Resources (CDWR). DWR Water Right - Net Amounts, <https://data.colorado.gov/Water/DWR-Water-Right-Net-Amounts/acsg-f33s/data> (2019).

18 Teeple, A. P. *Geophysics- and geochemistry-based assessment of the geochemical characteristics and groundwater-flow system of the U.S. part of the Mesilla Basin/Conejos-Médanos aquifer system in Doña Ana County, New Mexico, and El Paso County, Texas, 2010–12*. Scientific Investigations Report. Reston, VA, <https://doi.org/10.3133/sir20175028> (U. S. Geological Survey, 2017).

19 Driscoll, J. M. & Sherson, L. R. *Variability of surface-water quantity and quality and shallow groundwater levels and quality within the Rio Grande Project Area, New Mexico and Texas, 2009–13*. Scientific Investigations Report. Reston, VA, <http://dx.doi.org/10.3133/sir20165006> (U. S. Geological Survey, 2016).

20 Bureau of Land Management (BLM). National Surface Management Agency Area Polygons - National Geospatial Data Asset, <https://landscape.blm.gov/geoportal/catalog/main/portal.page> (2017).

21 Registro Agrario Nacional (RAN). Perimetrales de los núcleos agrarios certificados, <http://datos.ran.gob.mx/conjuntoDatosPublico.php> (2019).

22 Commission for Environmental Cooperation (CEC), Comisión Nacional de Áreas Naturales Protegidas (CONANP), Conservation Areas Reporting and Tracking System (CARTS), Ministère du developpement durable et de la lutte contre le changement climatique (Quebec-MDDELCC) & U.S. Geological Survey (USGS). Protected Areas of North America, 2017, <http://www.cec.org/tools-and-resources/north-american-environmental-atlas/map-files> (2017).

23 Homeland Infrastructure Foundation-Level Data (HIFLD). Canada and Mexico Border Crossings, <https://hifld-geoplatform.opendata.arcgis.com/datasets/canada-and-mexico-border-crossings> (2010).

24 Homeland Security Infrastructure Program (HSIP). Freedom Office of Border Patrol Sectors, <https://www.arcgis.com/home/item.html?id=e4c86699f8b84b6f9e5b26bf452323a8> (2017).

25 U.S. Customs and Border Protection (USCPB). Southwest Border Migration Apprehension Statistics. Fiscal years 2017 and 2018, <https://www.cbp.gov/newsroom/stats/usbp-sw-border-apprehensions> (2019).

26 U.S. Fish & Wildlife Service (USFWS). 2015 LCC Network Areas, <https://www.sciencebase.gov/catalog/item/55b943ade4b09a3b01b65d78> (2015).

27 U.S. Fish & Wildlife Service (USFWS). North American Joint Ventures, <https://ecos.fws.gov/ServCat/Reference/Profile/81433> (2017).

28 Food and Agriculture Organization (FAO). Hydrological basins in Central America (Derived from HydroSHEDS), <http://www.fao.org/geonetwork/srv/en/main.home> (2009).

29 Food and Agriculture Organization (FAO). Rivers in Central America (Derived from HydroSHEDS), <http://www.fao.org/geonetwork/srv/en/main.home> (2014).

30 U.S. Geological Survey (USGS). Ground Water Atlas of the United States. Aquifers vector digital data, <https://catalog.data.gov/dataset/aquifers> (2008).

31 Comisión Nacional del Agua (CONAGUA). Base Datos Bandas, <ftp://ftp.conagua.gob.mx/Bandas/Bases_Datos_Bandas> (2017).

32 Dieter, C. A. *et al.* Estimated Use of Water in the United States County-Level Data for 2015 (ver. 2.0, June 2018). U.S. Geological Survey data release, <https://doi.org/10.5066/F7TB15V5> (2018).

33 Instituto Nacional de Estadística y Geografía (INEGI). Censo Agrícola, Ganadero y Forestal 2007, <https://www.inegi.org.mx/programas/cagf/2007/default.html> (2009).

34 Texas Commission on Environmental Quality (TCEQ). WRAP Input files and GIS files by River Basin, [https://www.tceq.texas.gov/permitting/water_rights/wr_technical-resources/wam.html/#wrapinput](https://www.tceq.texas.gov/permitting/water_rights/wr_technical-resources/wam.html/%23wrapinput) (2013).

35 Comisión Nacional del Agua (CONAGUA). Tablero SINA: Registro Público de Derechos de Agua (Repda) / Volúmenes inscritos, <http://siga.conagua.gob.mx/REPDA/Menu/MenuKMZ.html> (2017).

36 Instituto Nacional de Estadística y Geografía (INEGI). Conjunto de datos vectoriales de la serie topográfica escala 1:1,000,000. Acueducto y Canal, <https://www.inegi.org.mx/app/biblioteca/ficha.html?upc=702825267643> (2000).

37 U.S. Census Bureau. Profile of General Population and Housing Characteristics: 1990, 2000, 2010, <https://factfinder.census.gov/faces/nav/jsf/pages/searchresults.xhtml?refresh=t> (2010).

38 Instituto Nacional de Estadística y Geografía (INEGI). Censos y Conteos de Población y Vivienda. Serie histórica censal e intercensal (1990-2010), <https://www.inegi.org.mx/programas/ccpv/cpvsh/> (2018).

39 Center for International Earth Science Information Network (CIESIN) - Columbia University. Gridded Population of the World, Version 4 (GPWv4): Population Density, Revision 11, <https://doi.org/10.7927/H49C6VHW> (2018).

40 U.S. Bureau of Economic Analysis. Annual Personal Income by State: 1969-2015, <https://apps.bea.gov/regional/Downloadzip.cfm> (2016).

41 Comisión Nacional para el Conocimiento y Uso de la Biodiversidad (CONABIO). Ingreso en México por municipio, 1990, 2000, 2010. Datos estadísticos del Instituto Nacional de Estadísitca y Geografía (INEGI), <http://www.conabio.gob.mx/informacion/gis/> (2012).

42 U.S. Department of Agriculture - National Agricultural Statistics Service (USDA-NASS). Census of Agriculture 2012 and 2007, <https://www.nass.usda.gov/AgCensus/index.php> (2014).

43 Center for International Earth Science Information Network (CIESIN) - Columbia University & Information Technology Outreach Services (ITOS) - University of Georgia. Global Roads Open Access Data Set, Version 1 (gROADSv1), <https://doi.org/10.7927/H4VD6WCT> (2013).

44 Instituto Nacional de Estadística y Geografía (INEGI), Natural Resources Canada (NRCan), U.S. Geological Survey (USGS) & Commission for Environmental Cooperation (CEC). North American Environmental Atlas - Railroads, 2009, <http://www.cec.org/tools-and-resources/north-american-environmental-atlas/map-files> (2010).

45 U.S. Environmental Protection Agency (EPA). Level III ecoregions of North America, <https://www.epa.gov/eco-research/ecoregions-north-america> (2010).

46 U.S. Environmental Protection Agency (EPA). Level III and IV ecoregions of the continental United States, <https://www.epa.gov/eco-research/level-iii-and-iv-ecoregions-continental-united-states> (2013).

47 U.S. Fish & Wildlife Service (USFWS). Threatened & Endangered Species Active Critical Habitat Report, <https://ecos.fws.gov/ecp/report/table/critical-habitat.html> (2019).

48 U.S. Fish & Wildlife Service (USFWS). National Wetlands Inventory. A System for Mapping Riparian Areas in the Western United States, <https://www.fws.gov/wetlands/Other/Riparian-Product-Summary.html> (2018).

49 U.S. Fish & Wildlife Service (USFWS). National Wetlands Inventory - Version 2 - Surface Waters and Wetlands Inventory, <https://www.fws.gov/wetlands/Data/Data-Download.html> (2018).

50 Comisión Nacional de Áreas Naturales Protegidas (CONANP). Sitios RAMSAR, <https://datos.gob.mx/busca/dataset/coberturas-para-manejadores-de-sig> (2015).

51 Comisión Nacional para el Conocimiento y Uso de la Biodiversida (CONABIO). Sitios de atención prioritaria para la conservación de la biodiversidad, <http://www.conabio.gob.mx/informacion/gis/> (2016).

52 Comisión Nacional para el Conocimiento y Uso de la Biodiversida (CONABIO). Sitios prioritarios para la restauración, <http://www.conabio.gob.mx/informacion/gis/> (2016).

53 Food and Agriculture Organization (FAO). The Digital Soil Map of the World, Version 3.6, <http://www.fao.org/geonetwork/srv/en/metadata.show?id=14116> (2003).

54 Sanchez, P. A. *et al.* Digital Soil Map of the World. *Science.* **325**, 680 (2009).

55 FAO/IIASA/ISRIC/ISS-CAS/JRC. Harmonized World Soil Database (version 1.1), <http://webarchive.iiasa.ac.at/Research/LUC/Products-Datasets/global-terrain-slope-download.html> (2009).

56 Canada Centre for Remote Sensing (CCRS)/Canada Centre for Mapping and Earth Observation (CCMEO) Natural Resources Canada (NRCan), Comisión Nacional para el Conocimiento y Uso de la Biodiversidad (CONABIO), Comisión Nacional Forestal (CONAFOR), Insituto Nacional de Estadística y Geografía (INEGI) & U.S. Geological Survey (USGS). 2010 Land Cover of North America at 30 meters. Edition 1.0, <http://www.cec.org/tools-and-resources/north-american-environmental-atlas/map-files> (2017).

57 U.S. Department of Agriculture - National Agricultural Statistics Service (USDA-NASS). 2008-2018 Cropland Data Layer, <https://nassgeodata.gmu.edu/CropScape/> (2019).

58 Instituto Nacional de Estadística y Geografía (INEGI). Conjunto de datos vectoriales de Uso del Suelo y Vegetación 2011, Escala 1:250,000, Serie V (Capa Unión), <https://www.inegi.org.mx/temas/usosuelo/> (2013).

59 Instituto Nacional de Estadística y Geografía (INEGI). Conjunto de datos vectoriales de Uso del Suelo y Vegetación 2014, Escala 1:250,000, Serie VI (Capa Unión), <https://www.inegi.org.mx/temas/usosuelo/> (2016).
