# Supplementary material for: A socio-environmental geodatabase for integrative research in the transboundary Rio Grande/Río Bravo basin
Source: Sci Data. 2020 Mar 6;7:80. doi: 10.1038/s41597-020-0410-1 (PMC7060182; doi:10.1038/s41597-020-0410-1)
Supplement: Supplementary file 3 — Supplementary Information 3 [file 41597_2020_410_MOESM3_ESM.docx]

### Supplementary Information 3: Acronyms

| **ATSWCD** | Association of Texas Soil and Water Conservation Districts |
| --- | --- |
| **BIA** | Bureau of Indian Affairs |
| **BJV** | Bird Joint Venture |
| **BLM** | Bureau of Land Management |
| **CACD** | Colorado Association of Conservation Districts |
| **CDWR or DWR** | Colorado Division of Water Resources |
| **CEAD** | Comisión de Agua del Estado de Durango |
| **CEAS** | Comisión Estatal de Aguas y Saneamiento de Coahuila |
| **CEAT** | Comisión Estatal del agua de Tamaulipas |
| **CENAPRED** | Centro Nacional de Prevención de Desastres |
| **CILA** | Comisión Internacional de Límites y Aguas |
| **CONABIO** | Comisión Nacional para el Conocimiento y Uso de la Biodiversidad |
| **CONAGUA** | Comisión Nacional del Agua |
| **CONANP** | Comisión Nacional de Áreas Naturales Protegidas |
| **CSCB** | Colorado State Conservation Board |
| **CWCB** | Colorado Water Conservation Board |
| **DEM** | Digital Elevation Model |
| **DOD** | Department of Defense |
| **DR** | Distrito de Riego (Irrigation District) |
| **EBID** | Elephant Butte Irrigation District |
| **EPWID#1** | El Paso Water Improvement District number 1 |
| **FAO** | Food and Agriculture Organization of United Nations |
| **FGDC CSDGM** | [Federal Geographic Data Committee Content Standard for Digital Geospatial Metadata](http://www.fgdc.gov/csdgmgraphical/index.html) |
| **GCD** | Groundwater Conservation District |
| **GMA** | Groundwater Management Area |
| **IBWC** | International Boundary and Water Commission |
| **INEGI** | Instituto Nacional de Estadística y Geografía |
| **JCAS** | Junta Central de Agua y Saneamiento in Chihuahua |
| **LCC** | Landscape Conservation Cooperative |
| **NACD** | National Association of Conservation Districts |
| **NALCMS** | North American Land Change Monitoring System |
| **NHD** | National Hydrographic Dataset |
| **NMDA** | New Mexico Department of Agriculture |
| **NMOSE** | New Mexico Office of the State Engineer |
| **NPS** | National Parks Services |
| **PGMA** | Priority Groundwater Management Area |
| **RAN** | Registro Agrario Nacional |
| **REPDA** | Registro Público de Derechos de Agua |
| **RGB** | Rio Grande/Río Bravo |
| **RGWCD** | Rio Grande Water Conservation District |
| **SADM** | Servicios de Agua y Drenaje de Monterrey |
| **SDG** | Sustainable Development Goals |
| **SIGA** | Sistema de Información Geográfica del Agua |
| **SJCP** | San Juan Chama Project |
| **SPS** | State Parks Services |
| **SWCDs** | Soil and Water Conservation Districts |
| **TAAP** | Transboundary Aquifer Assessment Program |
| **TCEQ** | Texas Commission on Environmental Quality |
| **TSSWCB** | Texas State Soil and Water Conservation Board |
| **TWDB** | Texas Water Development Board |
| **UN** | United Nations |
| **USACE** | U.S. Army Corps of Engineer |
| **USBR** | U.S. Bureau of Reclamation |
| **USDA-NASS** | U.S. Department of Agriculture – National Agricultural Statistics Services |
| **USDOT BTS** | U.S. Office of the Assistant Secretary for Research and Technology Bureau of Transportation Statistics |
| **USFS** | U.S. Forest Services |
| **USFWS** | U.S. Fish and Wildlife Services |
| **USGS** | U.S. Geological Survey |
| **WWF** | World Wildlife Fund |
